# Supplementary material for: A qualitative study comparing experiences of the surgical safety checklist in hospitals in high-income and low-income countries
Source: BMJ Open. 2013 Aug 14;3(8):e003039. doi: 10.1136/bmjopen-2013-003039 (PMC3752057; doi:10.1136/bmjopen-2013-003039)
Supplement: Author's manuscript [file bmjopen-2013-003039.draft_revisions.pdf]

Reality check for the surgical safety checklist? A qualitative study comparing high and low income countries

Journal:	*BMJ Open*
Manuscript ID:	bmjopen-2013-003039
Article Type:	Research
Date Submitted by the Author:	15-Apr-2013
Complete List of Authors:	Aveling, Emma-Louise; University of Leicester, Health Sciences<br>McCulloch, Peter; University of Oxford, Nuffield Surgery<br>DixonWoods, Mary; University of Leicester, Health Sciences
**Primary Subject Heading**:	Surgery
Secondary Subject Heading:	Qualitative research, Health services research
Keywords:	HEALTH SERVICES ADMINISTRATION & MANAGEMENT, International health services < HEALTH SERVICES ADMINISTRATION & MANAGEMENT, Protocols & guidelines < HEALTH SERVICES ADMINISTRATION & MANAGEMENT, Quality in health care < HEALTH SERVICES ADMINISTRATION & MANAGEMENT, QUALITATIVE RESEARCH, SURGERY

SCHOLARONE™ Manuscripts

For peer review only

15/04/2013

Reality check for the surgical safety checklist? A qualitative study comparing high and low income countries

Emma-Louise Aveling,[1] Peter McCulloch,[2] Mary Dixon-Woods [3*]

[1] Research Associate, Department of Health Sciences, University of Leicester, 22-28 Princess Road West, Leicester LE1 6TP

[2] Clinical Reader in Surgery, Nuffield Department of Surgical Science, University of Oxford

[3]Professor of Medical Sociology, Department of Health Sciences, University of Leicester, , 22-28 Princess Road West, Leicester LE1 6TP

*Corresponding author: Mary Dixon-Woods

Department of Health Sciences, University of Leicester, 22-28 Princess Road West, Leicester LE1 6TP

Email: md11@le.ac.uk; Tel: +44116 229 7262

For peer review only - http://bmjopen.bmj.com/site/about/guidelines.xhtml

15/04/2013

Article summary

ARTICLE FOCUS

- Bold claims have been made for the ability of the WHO surgical checklist to reduce surgical morbidity and mortality and improve patient safety.
- We aimed to identify and compare influences on checklist implementation and compliance in case study operating theatres in the UK and Africa.

KEY MESSAGES

- Consistent use, completion and fidelity of checklist deployment is not straightforward in any site, but appears higher in the UK compared with a low-income setting.
- Contrary to claims in early studies of the checklist, additional resources and changes to clinical systems may be needed to secure compliance with the checklist and its principles in low-income countries.
- Improvements in communication and quality of team interactions do not automatically follow the introduction of a surgical checklist. Indeed, the checklist may introduce new risks in low-income settings.

STRENGTHS AND LIMITATIONS

- Strengths: detailed, first-hand observations of the implementation of the surgical checklist in diverse settings; identification of key lessons of practical value in securing the benefits of the checklist and avoiding unintended consequences, especially in low-income countries
- Limitations: data on outcomes were unavailable; observations conducted at different stages of implementation; some evidence of observer effect; details of training were unreliable.

For peer review only

For peer review only - http://bmjopen.bmj.com/site/about/guidelines.xhtml

15/04/2013

ABSTRACT

Objective: Bold claims have been made for the ability of the WHO surgical checklist to reduce surgical morbidity and mortality and improve patient safety regardless of setting. Despite evidence of difficulties in checklist compliance in high income countries, little is known about how far the challenges faced by low income countries are the same or different. We aimed to identify and compare influences on checklist implementation and compliance in operating theatres in the UK and Africa.

Design: Ethnographic study involving observations, interviews, and collection of documents. Thematic analysis of data.

Setting: Operating theatres in one African university hospital and two UK university hospital

Participants: 112 hours of observations were undertaken. Interviews with 39 theatre and administrative staff were conducted.

Results: In both the UK and African settings, compliance was adversely affected by conflicts between the philosophy behind the checklist and the realities of local cultural and social context. Checklist compliance was considerably higher in a high-income UK setting, where it was supported by established structures and systems for accountability and audit, and was not significantly undermined by major resource constraints. Resentment of the checklist as unnecessary was noted in all settings. Hierarchical relationships were also a major barrier to implementation in all settings, but were more marked in the low-income African hospital. Introducing a checklist in a professional environment characterised by a lack of accountability and transparency could make staff feel jeopardised legally, professionally, and personally, and it encouraged them to make misleading records of what had actually been done.

Conclusion: Surgical checklist use is likely to be optimised, regardless of setting, when used as one tool in multi-faceted cultural and organisational programmes to strengthen patient safety. It cannot be assumed that the introduction of a checklist will automatically lead to improved communication and clinical processes.

For peer review only - http://bmjopen.bmj.com/site/about/guidelines.xhtml

15/04/2013

INTRODUCTION

Surgery provides immediate, transformative treatment for many conditions, usually in a single episode, and will remain a vital part of therapeutic strategy in all countries. Surgery is, unfortunately, also a major source of avoidable morbidity and mortality worldwide,[1] though substantial improvements can be achieved by reducing variation in the reliability of surgical care processes. Checklists are increasingly promoted as a way to deliver such reliability, following a pilot study of the World Health Organization's (WHO) Surgical Safety Checklist (Box 1) which reported important reductions in mortality and complications in surgical patients.[2] The checklist involves 19 separate items to be checked at three distinct points: the Sign In, before induction of anaesthesia; the Time Out, before skin incision; and the Sign Out, at the end of the procedure.

One important and unusual feature of the WHO checklist is its claim to universality. The checklist pilot study[2] was distinctive for its being conducted in high, middle and lower income settings, including one African hospital. It concluded that the checklist programme could "improve the safety of surgical patients in diverse clinical and economic environments" (p496). It also suggested that implementation was neither costly or lengthy, proposing that only two items – pulse oximetry and prophylactic antibiotics - would involve commitment of significant resources; both were reported to be available at all sites (including the low-income hospitals) in the study, though used inconsistently.

A second important feature of the WHO checklist is that it combines checks for technical items (such as administration of antibiotics and use of pulse oximeters) with other so called "non-technical" items (such as team introductions and confirmations of procedures) whose principal purpose is to promote aspects of teamwork, communication and situational awareness. The surgical checklist's inclusion of non-technical items was influenced by prior research demonstrating an association between team practices (e.g. communication practices) and improved safety processes and attitudes.[3, 4] Performing the checklist is also envisioned as a non-technical intervention in its own right: the checks are to be performed orally, and are, according to the checklist pilot study, "intentionally designed to create a collective awareness among surgical teams of whether safety processes are being completed" (p497).[2]

The potential for surgical safety checklists to improve safety and outcomes and generate substantial cost savings[5] has attracted global interest. In part owing to the WHO's Safe Surgery Saves Lives campaign, around 1800 institutions are now reported to be using the checklist worldwide.[6] However, the size of the reported effects on patient safety outcomes varies, possibly because of variability in compliance.[7-9] Compliance can be heuristically distinguished into three dimensions: use, completeness and fidelity. Use refers to whether the checklist is used at all; completeness the extent to which it is completed in full, without items being skipped; and fidelity the extent to which items are performed as intended, with items ticked as complete only when checks have genuinely been made, at the right time, and in communication with the whole team.

For peer review only - http://bmjopen.bmj.com/site/about/guidelines.xhtml

Implementing checklists and securing compliance necessarily requires an appreciation of sociocultural context.[10] Healthcare practitioners do not blindly follow procedures; rather, implementation often depends on workers' perceptions of effectiveness, relevance and rationale, their efforts to juggle competing professional, moral and social norms and demands, and the institutional and socio-economic contexts in which they work.[11, 12] However, the focus of the checklist pilot study[2] on reporting outcomes made it difficult to identify the implementation processes and characteristics of contexts likely to optimise the impact of checklist introduction. Understanding what they are and whether they differ across contexts is important to attempts to replicate positive effects in diverse settings. Studies of the checklist in high-income countries suggest that challenges to compliance are linked to organisational safety culture,[10] and may include hierarchical structures, dismissive attitudes, absence of key team members, and hurrying through checks.[13-15] Yet study of the checklist in low-income countries has been neglected. This is an important omission: complications and mortality following surgery in sub-Saharan Africa remain far higher than in developed countries[1] and almost 200 registered institutions in Africa are now reported to be using the checklist.[16] The specific challenges likely to be faced in such settings remain largely unknown. We aimed to identify and compare influences on checklist implementation and compliance in case study operating theatres in the UK and Africa.

METHODS

Our study design involved ethnographic case studies[17] in three hospitals: two in the UK (anonymised as Amfield and Tolgrave) and one (anonymised as Mbile) in a low-income sub-Saharan African country. The UK centres were two large university hospitals in major urban settings that were participating in a larger research project on culture and behaviour relating to patient safety. The African centre was a university hospital serving a mixed urban and rural population of approximately 4-5 million. All three hospitals had adopted, as official policy, a checklist based on the WHO template. The checklist had been mandated for use in all UK hospitals by the National Patient Safety Agency two years before our study; in Mbile, it was introduced one year before as part of an externally funded international collaboration to improve patient safety. Given the sensitivity of some findings, we do not provide further details that could make participating sites or individual staff identifiable. Ethical approvals were obtained for the study in the UK and in the African site.

Data collected across all three sites comprised: i) Semi-structured interviews with anaesthetists, surgeons, theatre staff, management and administrative staff covering experiences of checklist implementation, institutional context, and management of patient safety. Interviews were conducted with informed consent from participants and were recorded and translated (where required) and transcribed; ii) non-standardised observations and informal discussions with staff in operating rooms, recorded as field-notes in notebooks and later elaborated more fully; iii) collation of relevant documentation. ELA collected the data in two sites; a second observer (see acknowledgements) collected data in one of the UK sites. ELA analysed the data from all three sites.

Data were analysed thematically,[18] supported by Nvivo software, and guided but not constrained by sensitising concepts derived from the research questions.[19] A single coding framework was iteratively developed, refined and applied to all three data sets. Data were coded into basic-themes within three global categories - implementation barriers, facilitators and contextual characteristics.

For peer review only - http://bmjopen.bmj.com/site/about/guidelines.xhtml

In each category, codes were grouped into organising-themes, and patterns in different sites were identified, compared and contrasted, paying attention to interrelations between themes. In addition, we analysed data from each site to identify patterns of use, completeness and fidelity of checklist compliance.

RESULTS

We interviewed 39 staff: 19 in Mbile and 20 across the two UK hospitals (see Table 2). A total of 112 hours of observations were undertaken: 60 hours over two weeks in Mbile, 28 hours over four days in Amfield and 24 hours over three days in Tolgrave.

< TABLE 1 ABOUT HERE>

In all three sites, checklist implementation strategies included staff training, in situ demonstrations and awareness-raising at departmental meetings. In Mbile, training was assisted by two different international organisations, but surgeons were trained separately from other staff. This resulted in some mixed messages being circulated.

Though in all sites some staff attributed initial problems with checklist compliance to lack of awareness or understanding, most staff in both the UK and African hospitals could describe broadly how it was *intended* to be used. In all sites, many staff saw considerable value in the checklist. UK staff tended to emphasise prevention of rare but potentially catastrophic errors, such as wrong site surgery, and suggested checklist use could also improve communication and teamwork with “no hierarchy” (Amfield, anaesthetist).

> *We’re trying to prevent what are usually rare errors, rare mistakes, you know, the majority of the things on that checklist are done most of the time without the checklist, but every now and then [...] you forget to check if you’re operating on the right leg and not the left leg, and that’s rare, but on very rare occasions it then leads to a disaster. [Amfield, Anaesthetist, U021]*

> *It’s not something fluffy and friendly, it is actually functional and it is about all respecting one another [Tolgrave, Anaesthetist, U014]*

Mbile staff reported that few other protocols or standardised checks were in place in their hospital; some therefore welcomed the prompting and structure provided by the checklist. In contrast to the UK sites, they emphasised the checklist’s value in catching more common mistakes – such as forgetting prophylactic antibiotics – but did not mention a role for the checklist in undermining hierarchies or improving teamwork.

> *Whenever we do the checklist we identify that some things have been missed [Mbile, Anaesthetist, A005]*

Compliance

Nurses or theatre practitioners in all three hospitals had primary responsibility for initiating use of each checklist section, but differences in the way the checks were performed were evident. In UK hospitals, staff performed checks out loud in front of all team members who were present. In Mbile, checks were undertaken by an individual nurse, who ticked boxes based on his/her perceptions of

For peer review only - http://bmjopen.bmj.com/site/about/guidelines.xhtml

what had happened or quietly checked on a one-to-one basis whether designated individuals had remembered specific tasks.

> *[During the procedure] I see the nurse filling in the checklist, which he then puts on top of the cupboard. He doesn't speak to anyone in the process, except to talk quietly with one of the surgical residents. [Mbile fieldnotes]*

Though our study was not designed as a systematic audit, interviews and observations suggested that checklist use, completion, and fidelity, while not perfect, were more consistent in UK settings compared with Mbile. In the UK hospitals, the checklist was *used* in all procedures that we observed, documentation was fully *completed*, and interviewees reported internal audits showing checklist use in over 90% of procedures. *Fidelity* was more variable: in some instances teamwork was undermined by staff being distracted, dismissive, or absent during checks. During Time Out, staff often continued with other preparatory tasks, meaning it was not always the "moment of silence it's supposed to be" (nurse, Tolgrave). Sign In, supposed to be done before induction of anaesthesia, was often performed at the same time as the Time Out when the patient was already anaesthetised. Sign Out was often rushed or cursorily performed, though equipment counts were extremely thorough.

> *The scrub nurse and the student Operating Department Practitioner (ODP) are sort of removed, standing over by their metal trolley preparing some of the equipment and talking; they're not really listening to the rest of the group [surgeon, anaesthetist, ODP] who are crowded around patient (who's already anaesthetised), but they answer the questions when they're called to, and they do all the checks. [Amfield fieldnotes]*

> *At the end of the operation, just as the patient is being moved from the operating table onto a trolley, and they're waiting for him to wake up [..] the anaesthetist says "oh, we haven't done the sign out, oh, we should do the sign out". By this point the surgeon's already left the theatre [Amfield fieldnotes]*

In Mbile, *use* of the checklist was highly inconsistent: participants' estimates ranged from 'always' to 'hardly ever', and direct observations induced a Hawthorne effect, where checklist use increased from few procedures early in our observation period to all procedures by the final two days. When staff were under pressure due to staff shortages, emergencies, or lengths of shifts (up to 36 hours) not seen in the UK, the checklist was apt to be abandoned altogether.

> *Sometimes it's difficult to use this [checklist], due to staff overload, so sometimes if there is an emergency case, they may not fill it in. Rather than fill it in, they might just get the instruments for those guys because of the urgency of the case [Mbile, Theatre Nurse, A011]*

Completeness was both reported and observed to be highly variable in Mbile, with no improvement over the period of observations. *Fidelity* was also problematic in Mbile. Check-boxes were often ticked without the requisite information having been obtained or the tasks to which they referred undertaken, and the timing of checks was haphazard. Sometimes the nurse ticked the Sign-In and Time-Out checkboxes when the procedure was already underway, rendering them useless. Team introductions were never performed, yet the box was always ticked. Sometimes the Sign-Out boxes were ticked before the procedure was completed. This meant that equipment counts were ticked as complete when equipment was still in use, and specimens were recorded as correctly labelled before they had been removed from the patient. If equipment counts were performed at all in Mbile, they tended to be unreliable, completed in silence (not out loud as specified by the WHO

For peer review only - http://bmjopen.bmj.com/site/about/guidelines.xhtml

guidance) by a single nurse at variable points during the procedure using a form that did not list all relevant equipment.

> *[During the procedure] I check the checklist: the nurse had (at some point before the end of the operation) filled the entire thing in, including "specimen is correctly labelled" – even though the specimen-to-be was still inside the patient. [Mbile fieldnotes]*

> *The nurse fills in the back side of the form (the equipment count chart), even though all the equipment used in the operation has been cleared away, and completes the sign-out column of the checklist, without talking to anyone. [Mbile fieldnotes]*

Factors influencing checklist implementation

Some reasons for variable compliance were similar between the UK and African sites; others were different, and some problems that appeared to be the same had different underlying mechanisms. A shared barrier to consistent compliance across UK and the African settings was a perception among some that the checklist was futile, or that some checks (particularly the non-technical ones) were a waste of time.

> *The anaesthetist refuses to say his name. He says "I think we all know each other by now [...] it's ridiculous" [Amfield fieldnotes]*

> *No, no, no [we don't do introductions] We know each other! I know the anaesthetist, I know the nurse. [Mbile, Surgeon, A017]*

Perceptions of pointlessness of some elements of the checklist were partly based on the (demonstrably false) view among some staff that incidents the checklist was designed to prevent would never happen in their environments. In all sites the persuasive power of sentinel events – even very serious incidents (including, in Mbile, major operations on the wrong patients and a wrong site surgery in Amfield) – was variable.

> *I can clearly remember two occasions where the surgeon was just very dismissive and it was like, 'oh I'm not doing this, I don't need to do this, I'm not' you know... and literally just walked away. [Amfield, Operating Department Practitioner, U020]*

> *Even though training on the checklist was given for surgeons, they don't use it, they don't believe in this bit of paper, because mostly they said, 'we don't mistake the identity of the patient, it doesn't happen that we get the wrong patient' [Mbile, Theatre nurse, A012]*

Differences in factors influencing compliance, and their underlying mechanisms, derived from the very different contexts of implementation. Perhaps the most obvious difference between the settings concerned material resources. In all settings, the technical items were broadly accepted as established good practice. In the UK hospitals, equipment and medicines were available to address these items. Mbile's infrastructure was very different. The electricity supply, for example, was unreliable; even basic equipment, such as gauze, was not reliably available. Such resource shortages meant that it was not always possible to comply with the technical items on the checklist, yet nurses often ticked the boxes nonetheless. For example, there were no surgical markers at Mbile hospital, but documentary analysis showed the question 'is the site marked?' was always ticked 'yes'. The box for prophylactic antibiotics was also among those most consistently completed ('yes' or 'not

For peer review only - http://bmjopen.bmj.com/site/about/guidelines.xhtml

For peer review only

applicable' were usually ticked), but, with only a limited range of antibiotics available and no hospital policy regarding administration of prophylactic antibiotics, doctors sometimes delayed antibiotic use in case of subsequent post-operative infections. There were too few pulse oximeters available for the number of operations being performed. Anaesthetists were aware of the value of pulse oximeters, but were often unable to access one; absence of the device rarely led to cancellation of an operation.

> *We don't have too many types of antibiotics [...] if we have given [the patient] prophylactic antibiotics and organisms have already developed some resistance to the antibiotics, how are we going to treat this guy if he develops some type of infection later on? That is the problem [...] but we really don't have single view in this issue, I mean, some people give antibiotics, some people don't give, it's not a standardised policy [Mbile, Surgeon, A019]*

> *In set-ups like ours, for example if there is no pulse oximetry, [..] if you consider it as a must we're going to lose many people because often we don't have this pulse oximetry. So if you say now I'm not going to anaesthetise a patient because I have no pulse oximeter definitely we will lose many patients that could have been helped if you operate on them without pulse oximeter. [Mbile, Anaesthetist, A002]*

The non-technical items on the checklist were less dependent on material resources, yet they were much more likely to attract criticism and scepticism from staff. Some staff in all hospitals (UK and African) objected to the performance of the checklist in its entirety; more often, specific items were the subject of complaint. In the UK sites, a recurring objection was that the checklist duplicated pre-existing systems and checks, including, for example, long-standing equipment counting procedures. Perceptions of "duplicative" or "useless" checks contributed to the view of some in all settings that the checklist was an illegitimate bureaucratic intrusion, and resentment towards it intensified during busy periods, emergencies or staff shortages. The most deeply felt and repeatedly vocalised objections came from surgeons. Given strongly hierarchical team dynamics in both the UK and Africa, surgeons' behaviour and attitudes in relation to the checklist were highly influential, making it difficult for others to directly challenge defiant or dismissive surgeons.

> *I think it's difficult because there is a hierarchy within the theatre complex, or within the medical profession, full stop and the theatre staff might feel intimidated by some of the environments they are in. Some of them are forceful enough and have a voice, but others are quite timid and probably quite reluctant to actually speak up. [Tolgrave, Anaesthetist, U012]*

> *The surgeon may not volunteer to cooperate to fill that checklist. [...] The nurse may stop her work, or may do it without filling the checklist. It may depend on the confidence of the nurse. [Mbile, Theatre Nurse, A013]*

Despite these objections, use of the checklist was high in the UK hospitals. Staff in these hospitals had been highly sensitised to issues of patient safety through exposure to their institutions' policies and, though they did not see all patient safety interventions as positive, they felt that they had "a microscope on us about patient safety" (Tolgrave, nurse). Audit and feedback of data on checklist use and relevant outcomes (e.g. infection rates) were used to support checklist implementation and were tied to sanctions, which could include disciplinary action against staff who refused to comply.

For peer review only - http://bmjopen.bmj.com/site/about/guidelines.xhtml

You know I do not mind being part of any audit at all because I think it's great, we won't find anything or improve anything unless we do. [Tolgrave, ODP, U001]

I guess really just the fact that we have got a clearly focused campaign that says we will improve quality and safety, it's making everyone step up to the plate and consider what they are doing and being audited on it. [Tolgrave, Anaesthetist,U014]

We have to be careful because you know, it's in our registration as well to follow this protocol isn't it, so [even] if the surgeon is not keen to do it, we have to tell them, you have to stop and do it [Tolgrave, ODP, U 008]

These audits were not, however, perfect instruments, not least because they generally failed to capture *how* checklists were being used. Beyond these formal arrangements, some local leaders acted as champions for the checklist. Surgeons, anaesthetists and senior nurses in both UK settings took on this role, and were important in leading by example and supporting junior or non-surgical staff when there was "push-back".

We are there to iron out any problems, or if anybody gives them grief we can go in and fire it back if needed. [Tolgrave, Site coordinator, 002]

In Mbile, introducing the checklist involved much more far-reaching change than in the UK. The absence of an established tradition of equipment counts, for example, meant that the checklist was a much more disruptive and demanding intervention in the African setting. Items that were non-controversial in a UK setting, such as documenting patient consent, had different meaning in a context where patients often lacked literacy and surgery was considered a privilege. The surgical checklist was one of the first explicit patient safety interventions introduced in Mbile. Staff had little prior exposure to the ideas and principles of the science of safety, and systems to support patient safety were poorly developed. The hospital lacked an established culture of audit; there was little routine collection of clinical data and staff had no clear sense of the impact of the checklist on patient safety. There was no monitoring of checklist use, and no consequences for non-compliance.

I asked every department [if they want to do an audit] and they have not volunteered […] I think they're busy. It is not familiar, clinical audit. Most health workers don't know what clinical audit means. [Mbile, Administrator, A001]

We really don't have the real number [for infection rates], how many patients have been affected, because we haven't applied those measurements, we really don't know [Mbile, Surgeon, A020]

It's ok, nothing happens. For those who don't fill in the checklist there is no problem, no consequence. [Mbile, Theatre nurse, A011]

Team dynamics were especially challenging in Mbile. Though local championing of the checklist was not absent, it was not highly visible either. Surgeons were the only doctors in the room: anaesthetists were not physicians, but held a BSc in anaesthesia. Wider societal hierarchies and cultural norms, including those relating to age, gender and education, resulted in particularly steep authority gradients. Nurses were often female and younger than the (exclusively) male surgeons, and had been socialised to be deferential and submissive. The working environment was described as lacking in transparency and accountability, particularly in relation to staff dismissals or promotions.

For peer review only - http://bmjopen.bmj.com/site/about/guidelines.xhtml

There were complaints that there is impartial [unfair] treatment between people. This is one of the problems from the managerial aspect. [Mbile, Anaesthetist,A 002]

The external socio-legal context in Mbile was one that staff felt was at best unreliable. At worst it was seen corrupt and unjust, offering little protection to vulnerable, low-status individuals. Interviews suggested that challenging those in authority could therefore feel very risky and have unpredictable results, perhaps damaging career prospects or even worse (Box 2). In observations, challenge over checklist use was very rarely witnessed. The checklist thus introduced new, unintended risks for staff.

<Box 2 about here>

DISCUSSION

The WHO's implementation manual identifies two purposes for the surgical checklist: ensuring consistency in patient safety and introducing (or maintaining) a culture that values achieving it.[20] Our study suggests that hospitals in both high and low-income countries may experience challenges in delivering on these aims. An important finding is the extent to which hospitals in both low and high-income countries encountered the same obstacles in implementing the checklist; the key differences related to contexts. Material contexts were perhaps most vividly distinct: resource shortages meant that it was often impossible to comply with the checklist's technical requirements in Mbile. Resource and infrastructure constraints in low-income countries have been well described previously,[21-23] suggesting that the problems in our African study hospital were unlikely to be unique. Many of the non-technical items on the checklist did not require additional material resources, but rather changes in communication practices and teamwork. Hierarchical team dynamics undermining of safety were evident in all three hospitals in our study, but were more pronounced in the African setting. Our findings raise questions about the claim in the pilot study of the checklist[2] that few additional resources or changes to clinical systems would be needed to implement the checklist. Our findings also challenge the assumption that improvements in communication and team interactions automatically follow the introduction of a checklist.[2, 24] Contrary to the pilot study hypothesis, introduction of the checklist did not "signify" "associated cultural changes"[2] (p.493); rather, our study points to a much more complex interrelation between checklist procedures, context, 'culture' and behavioural changes.

Contextual differences meant that Mbile had much more to do to implement the checklist, and lacked features that supported implementation in the UK sites. Local policies, institutional focus and support, well-established audit and data collection systems, clear lines of accountability with consequences for staff and hospitals for non-compliance were all more pronounced (if not always perfect) in the UK hospitals than their African counterpart. They were important indirectly, by signalling the institutional priority given to patient safety,[14] and directly (e.g. disciplinary action). Despite these advantages, the UK sites also suffered from significant barriers to implementation, with evidence of persisting problems of fidelity, audits that focused too much on documentation of box-ticking, and resentment of perceived top-down initiatives.

Our study produced some disquieting evidence that poor checklist implementation in low-income settings might not only fail to reduce patient safety risks, but also *introduce* new risks for staff

For peer review only - http://bmjopen.bmj.com/site/about/guidelines.xhtml

For peer review only

and/or patients. Previous work has also shown that even in well-resourced settings, checklists may not consistently deliver positive effects, and may potentially produce some paradoxical or harmful effects including inhibition of team processes.[3, 25] These may be even more consequential in a low income setting. The style of checks in Mbile limited improvements in team communication, and was directly contrary to the design intent of creating collective awareness. The authoritarian status of the surgeon elite contributed to these unhelpful features of team dynamics, which were exacerbated by wider societal hierarchies and a lack of transparency in institutional processes. Though unhelpful team dynamics were also sometimes evident in the UK hospitals, introducing a checklist in a professional environment characterised by a lack of accountability and transparency raised additional ethical concerns in Mbile: it made staff feel jeopardised legally, professionally, and personally, and it encouraged them to make misleading records of what had actually been done. In this situation the benefits for patient safety were unclear, especially as no audit data were available to establish outcomes.

Though the volume of research on patient safety in low-income countries is now increasing,[26, 27] little is known about which kinds of strategies are most effective in addressing patient safety issues in these settings.[28] Our study suggests that explicit interventional focus on improving team dynamics and communication may be even more critical in African healthcare settings than high-income countries. Professional groups – in whatever setting - should be trained together on the checklist, not separately as occurred in Mbile. Additional interventional focus on relevant clinical systems may also be necessary. The critical role of local leads in motivating the adoption of new ideas[29] means that particular efforts must be made to secure buy-in from surgeons and to ensure that junior or non-medical staff are assured of institutional support. While high-level institutional strategies were seen by some as misdirected or problematic in UK settings, the comparison with Mbile nonetheless underscores the value of all front-line staff having high level institutional support and clear leadership for their efforts to improve safety.

Our study has a number of limitations. It did not include data on outcomes. Mbile had only been implementing the checklist for one year, in contrast to two years in UK hospitals, so was at a different stage of implementation. Despite the importance of training,[30] the precise nature of what was provided was unclear, due to unreliable (and in some cases conflicting) accounts of events. A clear observer effect was found in Mbile, and may also have occurred in UK hospitals. Observations over a longer period in all sites might have identified more influences on checklist compliance. Nonetheless, our study has yielded important lessons for implementing surgical checklists (Box 3).

Our work demonstrates that assumptions about context are encoded into patient safety tools. Optimising checklist use requires careful analysis of these assumptions, and of the extent to which these match local set-ups or require the introduction of new systems and practices. It also demonstrates that changes in teamwork practices will not automatically follow checklist introduction, even where non-technical items are included, but rather require explicit, interventional focus. Checklists are neither a 'quick-fix' nor a tool that can be effectively implemented in isolation; in resource-constrained settings, they are especially unlikely to be free of costs and risks. Safety checklists are most likely to be effective and sustainable when implemented as part of broader, multi-faceted programs addressing social, behavioural, logistical and organisational issues,[10, 31-33]

For peer review only - http://bmjopen.bmj.com/site/about/guidelines.xhtml

where there is strong institutional focus on patient safety, multi-disciplinary leadership, monitoring systems in place, and consequences at all levels for non-compliance.

<Box 3 about here>

For peer review only

For peer review only - http://bmjopen.bmj.com/site/about/guidelines.xhtml

Role/ Profession	Mbile (African hospital)	Amfield and Tolgrave (UK hospitals)
Anaesthetists	4	4
Surgeons	6	3
Managers/administrators	4	4
Theatre staff	5	9
Total	***19***	***20***

Table 1. Number of individual interviews by profession in African and UK sites

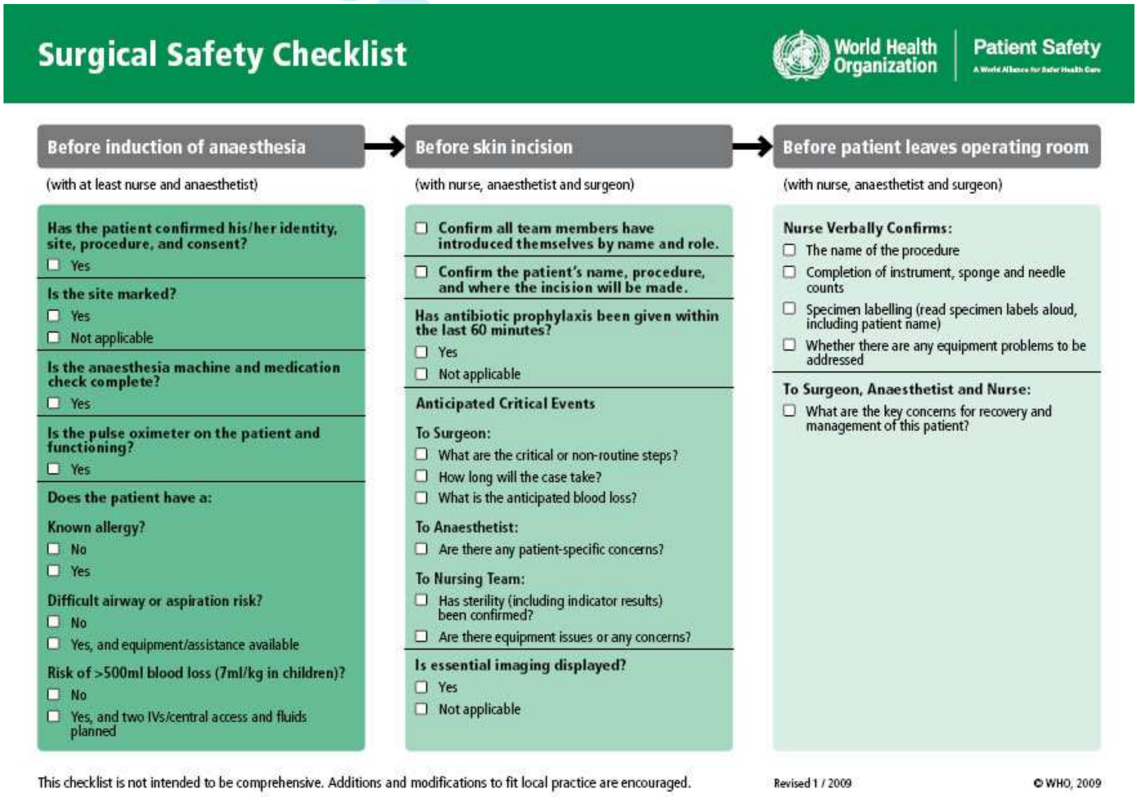
Surgical Safety Checklist

World Health Organization | Patient Safety

Before induction of anaesthesia

(with at least nurse and anaesthetist)

Has the patient confirmed his/her identity, site, procedure, and consent?
☐ Yes

Is the site marked?
☐ Yes
☐ Not applicable

Is the anaesthesia machine and medication check complete?
☐ Yes

Is the pulse oximeter on the patient and functioning?
☐ Yes

Does the patient have a:

Known allergy?
☐ No
☐ Yes

Difficult airway or aspiration risk?
☐ No
☐ Yes, and equipment/assistance available

Risk of >500ml blood loss (7ml/kg in children)?
☐ No
☐ Yes, and two IVs/central access and fluids planned

Before skin incision

(with nurse, anaesthetist and surgeon)

☐ **Confirm all team members have introduced themselves by name and role.**

☐ **Confirm the patient's name, procedure, and where the incision will be made.**

Has antibiotic prophylaxis been given within the last 60 minutes?
☐ Yes
☐ Not applicable

Anticipated Critical Events

To Surgeon:
☐ What are the critical or non-routine steps?
☐ How long will the case take?
☐ What is the anticipated blood loss?

To Anaesthetist:
☐ Are there any patient-specific concerns?

To Nursing Team:
☐ Has sterility (including indicator results) been confirmed?
☐ Are there equipment issues or any concerns?

Is essential imaging displayed?
☐ Yes
☐ Not applicable

Before patient leaves operating room

(with nurse, anaesthetist and surgeon)

Nurse Verbally Confirms:
☐ The name of the procedure
☐ Completion of instrument, sponge and needle counts
☐ Specimen labelling (read specimen labels aloud, including patient name)
☐ Whether there are any equipment problems to be addressed

To Surgeon, Anaesthetist and Nurse:
☐ What are the key concerns for recovery and management of this patient?

This checklist is not intended to be comprehensive. Additions and modifications to fit local practice are encouraged.

Revised 1 / 2009

© WHO, 2009

Box 1: WHO Surgical Safety Checklist (permission to use Figure currently awaited)

For peer review only - http://bmjopen.bmj.com/site/about/guidelines.xhtml

A patient admitted for cholecystectomy suffered hypoxic brain injury and died following surgery. Subsequently, two staff members (not the surgeon) were threatened with guns by the patient's family, who said the surgical team had killed the patient. The two staff members were later arrested and criminal charges brought against one of them. One of the questions asked during the investigation was whether a pulse oximeter had been used. It had not: according to staff, no pulse oximeter was available for use, even though the checklist requiring use of this equipment was official hospital policy. Adoption of the checklist by the hospital, without adequate provision of the resources necessary for compliance, had left low-status front-line staff more socially and legally vulnerable, as it could be argued that they had failed to comply with hospital policy. Moreover, at all levels of the institution there was a great deal of uncertainty about staff rights and their employers' responsibilities in such circumstances. The accused staff were not provided with any legal representation for some weeks.

Box. 2: Description of events following an adverse event in Mbile, illustrative of the wider socio-legal context.

For peer review only - http://bmjopen.bmj.com/site/about/guidelines.xhtml

15/04/2013

- Checklist implementation will be optimised when part of a broader, multi-faceted cultural and organisational programme to strengthen patient safety
- Introduction of the checklist does not automatically lead to changes in teamwork and communication, despite the inclusion of non-technical items
    - Improvements in communication and teamwork require direct interventional focus
    - Whole team training (rather than separate training for different professional groups) is important
    - Hierarchical dynamics within the operating theatre may be particularly prominent in some low-income contexts, exacerbated by the wider socio-legal context
- Assumptions about implementation contexts (e.g. resources, policies, systems in place) encoded within the checklist require careful scrutiny
    - Performance of the checklist may not be the only change(s) required
    - In resource-constrained settings especially, existing systems and resources may need to be strengthened for the checklist to be used with completeness and fidelity
- Local leadership and championing of the checklist is important and needs to:
    - Include support from the highest institutional level
    - Include senior staff, particularly senior surgeons
    - Include leaders from all professional groups
    - Be visible in theatres
- Monitoring (including process and impact measures) must be in place and fed back to staff
- Poor compliance (use, completeness and/or fidelity) may not only fail to strengthen surgical safety - it may also introduce risks for staff and/or patients in some environments

Box 3. Lessons for checklist implementation in high- and low-income country settings

For peer review only - http://bmjopen.bmj.com/site/about/guidelines.xhtml

Declarations

Acknowledgements: Our sincere thanks to the staff of all three hospitals for their time and participation in the study. Our thanks to Joel Minion, who collected the data from one of the UK sites. We would also like to thank Dr Wynne Aveling for the clinical expertise he offered during data collection.

Ethics: Ethical approval was granted by the Leicestershire, Northamptonshire and Rutland Research Ethics Committee (number 10/H0406/77), and the Institutional Ethical Review Board for research in the African site. All participants gave informed consent.

Funding: We thank the Higher Education Innovation Fund Impact Award; Wellcome Trust Senior Investigator Award WT097899MA; and the Department of Health Policy Research Programme (reference number 0770017) for providing funding for this study.

Role of sponsor: The funders did not have a role in study design; in the collection, analysis, and interpretation of data; in the writing of the report or the decision to submit the article for publication. The research was conducted independently of the funders.

Contributorship: ELA and MDW jointly conceived and designed the study with support from PM. ELA negotiated access, recruited participants and conducted the fieldwork in one UK site and the African site. ELA analysed the data from all three sites. ELA and MDW conducted the literature review, with additional suggestions from PM. ELA and MDW led the writing of the manuscript. PM contributed clinical expertise. All authors contributed to interpretation of the findings, contributed to drafting the manuscript and approved the final version. ELA is the guarantor.

Data access: ELA had full access to all of the data in the study and can take responsibility for the integrity of the data and the accuracy of the data analysis. MDW had full access to the data from the UK sites and the opportunity to access the African data. For ethical reasons full data were not shared with PM.

Data sharing: No additional data available.

Competing interest declaration: All authors have completed the Unified Competing Interest form at www.icmje.org/coi_disclosure.pdf (available on request from the corresponding author) and declare that (1) ELA, MDW, PM] have no relationships with [any companies] that might have an interest in the submitted work in the previous 3 years; (2) their spouses, partners, or children have no financial relationships that (3) ELA, MDW, PM have no non-financial interests that may be relevant to the submitted work.

For peer review only - http://bmjopen.bmj.com/site/about/guidelines.xhtml

15/04/2013

The Corresponding Author has the right to grant on behalf of all authors and does grant on behalf of all authors, a worldwide licence (http://www.bmj.com/sites/default/files/BMJ%20Author%20Licence%20March%202013.doc) to the Publishers and its licensees in perpetuity, in all forms, formats and media (whether known now or created in the future), to i) publish, reproduce, distribute, display and store the Contribution, ii) translate the Contribution into other languages, create adaptations, reprints, include within collections and create summaries, extracts and/or, abstracts of the Contribution and convert or allow conversion into any format including without limitation audio, iii) create any other derivative work(s) based in whole or part on the on the Contribution, iv) to exploit all subsidiary rights to exploit all subsidiary rights that currently exist or as may exist in the future in the Contribution, v) the inclusion of electronic links from the Contribution to third party material where-ever it may be located; and, vi) licence any third party to do any or all of the above. All research articles will be made available on an Open Access basis (with authors being asked to pay an open access fee—see http://www.bmj.com/about-bmj/resources-authors/forms-policies-and-checklists/copyright-open-access-and-permission-reuse). The terms of such Open Access shall be governed by a Creative Commons licence—details as to which Creative Commons licence will apply to the research article are set out in our worldwide licence referred to above.

For peer review only

For peer review only - http://bmjopen.bmj.com/site/about/guidelines.xhtml

15/04/2013

References

1. Bainbridge D, Martin J, Arango M, Cheng D, Evidence-based Peri-operative Clinical Outcomes Research (EPiCOR) Group. Perioperative and anaesthetic-related mortality in developed and developing countries: a systematic review and meta-analysis. *Lancet* 2012; Sep 22;380(9847):1075-81.

2. Haynes AB, Weiser TG, Berry WR, Lipsitz SR, Breizat AHS, Dellinger EP, et al. A surgical safety checklist to reduce morbidity and mortality in a global population. *N Engl J Med* 2009;260:491-9.

3. Pronovost P, Needham D, Berenholtz S, Sinopoli D, Chu H, Cosgrove S, et al. An intervention to decrease catheter-related bloodstream infections in the ICU. *N Engl J Med* 2006;355(26):2725-32.

4. Lingard L, Regehr G, Orser B, Reznick R, Baker GR, Doran D, et al. Evaluation of a preoperative checklist and team briefing among surgeons, nurses, and anesthesiologists to reduce failures in communication. *Archives of Surgery* 2008;143(1):12-7.

5. Leonard M, Graham S, Bonacum D. The human factor: The critical importance of effective teamwork and communication in providing safe care. *Quality and Safety in Health Care* 2004;13(SUPPL. 1):i85-90.

6. Dixon-Woods M, Suokas A, Pitchforth E, Tarrant C. An ethnographic study of classifying and accounting for risk at the sharp end of medical wards. *Soc Sci Med* 2009; Aug;69(3):362-9.

7. McCarthy JC, Wright PC, Monk AF, Watts LA. Concerns at work: Designing useful procedures. *Human-Computer Interaction* 1998;13:433-57.

8. Semel ME, Resch S, Haynes AB, Funk LM, Bader A, Berry WR, et al. Adopting a surgical safety checklist could save money and improve the quality of care in U.S. hospitals. *Health Aff (Millwood)* 2010; Sep;29(9):1593-9.

9. Surgical Safety Web Map. Available at: http://maps.cga.harvard.edu:8080/Hospital/. Accessed 07/12, 2012.

10. van Klei WA, Hoff RG, van Aarnhem EE, Simmermacher RKJ, Regli LPE, Kappen TH, et al. Effects of the introduction of the WHO "Surgical Safety Checklist" on in-hospital mortality: A cohort study. *Annals of Surgery* 2012;255(1):44-9.

11. Fourcade A, Blache J-, Grenier C, Bourgain J-, Minvielle E. Barriers to staff adoption of a surgical safety checklist. *BMJ Quality and Safety* 2012;21(3):191-7.

12. Levy SM, Senter CE, Hawkins RB, Zhao JY, Doody K, Kao LS, et al. Implementing a surgical checklist: More than checking a box. *Surgery (United States)* 2012;152(3):331-6.

13. Bosk CL, Dixon-Woods M, Goeschel CA, Pronovost PJ. The art of medicine. Reality check for checklists. *The Lancet* 2009;374(9688):444-5.

For peer review only

For peer review only - http://bmjopen.bmj.com/site/about/guidelines.xhtml

14. Vats A, Vincent CA, Nagpal K, Davies RW, Darzi A, Moorthy K. Practical challenges of introducing WHO surgical checklist: UK pilot experience. *BMJ* 2010;340.

15. Conley DM, Singer SJ, Edmondson L, Berry WR, Gawande AA. Effective surgical safety checklist implementation. *J Am Coll Surg* 2011;212(5):873-9.

16. Whyte S, Cartmill C, Gardezi F, Reznick R, Orser BA, Doran D, et al. Uptake of a team briefing in the operating theatre: A Burkean dramatistic analysis. *Social Science and Medicine* 2009;69(12):1757-66.

17. Safe Surgery in Africa. Available at: http://www.who.int/patientsafety/news/sssl_articles/en/index.html. Accessed 7/7, 2012.

18. Dixon-Woods M. What can ethnography do for quality and safety in health care?. *Qual Saf Health Care* 2003; Oct;12(5):326-7.

19. Attride-Stirling J. Thematic networks: An analytic tool for qualitative research. *Qualitative Research* 2001;1(3):385,405; 385.

20. Charmaz K. *Constructing grounded theory: A practical guide through qualitative analysis.* London: Sage; 2006.

21. Hsia RY, Mbembati NA, MacFarlane S, Kruk ME. Access to emergency and surgical care in sub-Saharan Africa: The infrastructure gap. *Health Policy Plan* 2012;27(3):234-44.

22. Deckelbaum DL, Ntakiyiruta G, Liberman AS, Razek T, Kyamanywa P. Augmenting surgical capacity in resource-limited settings. *The Lancet* 2011;.

23. Funk LM, Weiser TG, Berry WR, Lipsitz SR, Merry AF, Enright AC, et al. Global operating theatre distribution and pulse oximetry supply: An estimation from reported data. *The Lancet* 2010;376(9746):1055-61.

24. De Vries EN, Prins HA, Crolla RMPH, Den Outer AJ, Van Andel G, Van Helden SH, et al. Effect of a comprehensive surgical safety system on patient outcomes. *N Engl J Med* 2010;363(20):1928-37.

25. Whyte S, Lingard L, Espin S, Baker GR, Bohnen BA, Orser B, et al. Paradoxical effects of interprofessional briefings on OR team performance. *Cognition, Technology and Work* 2008;10(4):287-94.

26. Wilson D. Who Guards the Guardians? Ian Kennedy, Bioethics and the 'Ideology of Accountability' in British Medicine. *Social History of Medicine* 2011; July 01;.

27. Aiken AM, Karuri DM, Wanyoro AK, Macleod J. Interventional studies for preventing surgical site infections in sub-Saharan Africa - A systematic review. *International Journal of Surgery* 2012;10(5):242-9.

28. Siddiqi K, Newell J, Robinson M. Getting evidence into practice: What works in developing countries?. *International Journal of Quality in Health Care* 2005;17:447-54.

For peer review only

For peer review only - http://bmjopen.bmj.com/site/about/guidelines.xhtml

29. Edmondson AC. Speaking up in the operating room: How team leaders promote learning in interdisciplinary action teams. *Journal of Management Studies* 2003;40(6):1419-52.

30. McCulloch P, Rathbone J, Catchpole K. Interventions to improve teamwork and communications among healthcare staff. *British Journal of Surgery* 2011;98(4):469-79.

31. Ider B-, Adams J, Morton A, Whitby M, Muugolog T, Lundeg G, et al. Using a checklist to identify barriers to compliance with evidence-based guidelines for central line management: A mixed methods study in Mongolia. *International Journal of Infectious Diseases* 2012;16(7):e551-7.

32. Kotagal M, Lee P, Habiyakare C, Dusabe R, Kanama P, Epino HM, et al. Improving quality in resource poor settings: Observational study from rural Rwanda. *BMJ* 2009;339:1311-3.

33. Walker IA, Merry AF, Wilson IH, McHugh GA, O'Sullivan E, Thoms GM, et al. Global oximetry: An international anaesthesia quality improvement project. *Anaesthesia* 2009;64(10):1051-60.

1. Bainbridge D, Martin J, Arango M, Cheng D, Evidence-based Peri-operative Clinical Outcomes Research (EPiCOR) Group. Perioperative and anaesthetic-related mortality in developed and developing countries: a systematic review and meta-analysis. *Lancet* 2012; Sep 22;380(9847):1075-81.

2. Haynes AB, Weiser TG, Berry WR, Lipsitz SR, Breizat AHS, Dellinger EP, et al. A surgical safety checklist to reduce morbidity and mortality in a global population. *N Engl J Med* 2009;260:491-9.

3. Lingard L, Regehr G, Orser B, Reznick R, Baker GR, Doran D, et al. Evaluation of a preoperative checklist and team briefing among surgeons, nurses, and anesthesiologists to reduce failures in communication. *Archives of Surgery* 2008;143(1):12-7.

4. Leonard M, Graham S, Bonacum D. The human factor: The critical importance of effective teamwork and communication in providing safe care. *Quality and Safety in Health Care* 2004;13(SUPPL. 1):i85-90.

5. Semel ME, Resch S, Haynes AB, Funk LM, Bader A, Berry WR, et al. Adopting a surgical safety checklist could save money and improve the quality of care in U.S. hospitals. *Health Aff (Millwood)* 2010; Sep;29(9):1593-9.

6. Surgical Safety Web Map. Available at: http://maps.cga.harvard.edu:8080/Hospital/. Accessed 07/12, 2012.

7. van Klei WA, Hoff RG, van Aarnhem EE, Simmermacher RKJ, Regli LPE, Kappen TH, et al. Effects of the introduction of the WHO "Surgical Safety Checklist" on in-hospital mortality: A cohort study. *Annals of Surgery* 2012;255(1):44-9.

8. Fourcade A, Blache J-, Grenier C, Bourgain J-, Minvielle E. Barriers to staff adoption of a surgical safety checklist. *BMJ Quality and Safety* 2012;21(3):191-7.

9. Levy SM, Senter CE, Hawkins RB, Zhao JY, Doody K, Kao LS, et al. Implementing a surgical checklist: More than checking a box. *Surgery (United States)* 2012;152(3):331-6.

For peer review only

10. Bosk CL, Dixon-Woods M, Goeschel CA, Pronovost PJ. The art of medicine. Reality check for checklists. *The Lancet* 2009;374(9688):444-5.

11. Dixon-Woods M, Suokas A, Pitchforth E, Tarrant C. An ethnographic study of classifying and accounting for risk at the sharp end of medical wards. *Soc Sci Med* 2009; Aug;69(3):362-9.

12. McCarthy JC, Wright PC, Monk AF, Watts LA. Concerns at work: Designing useful procedures. *Human-Computer Interaction* 1998;13:433-57.

13. Vats A, Vincent CA, Nagpal K, Davies RW, Darzi A, Moorthy K. Practical challenges of introducing WHO surgical checklist: UK pilot experience. *BMJ* 2010;340.

14. Conley DM, Singer SJ, Edmondson L, Berry WR, Gawande AA. Effective surgical safety checklist implementation. *J Am Coll Surg* 2011;212(5):873-9.

15. Whyte S, Cartmill C, Gardezi F, Reznick R, Orser BA, Doran D, et al. Uptake of a team briefing in the operating theatre: A Burkean dramatistic analysis. *Social Science and Medicine* 2009;69(12):1757-66.

16. Safe Surgery in Africa. Available at: http://www.who.int/patientsafety/news/sssl_articles/en/index.html. Accessed 7/7, 2012.

17. Dixon-Woods M. What can ethnography do for quality and safety in health care?. *Qual Saf Health Care* 2003; Oct;12(5):326-7.

18. Attride-Stirling J. Thematic networks: An analytic tool for qualitative research. *Qualitative Research* 2001;1(3):385,405; 385.

19. Charmaz K. *Constructing grounded theory: A practical guide through qualitative analysis.* London: Sage; 2006.

20. WHO World Alliance for Patient Safety. Implementation manual WHO Surgical Safety Checklist (first edition). ; 2008. Report No.: WHO/IER/PSP/2008.05.

21. Hsia RY, Mbembati NA, MacFarlane S, Kruk ME. Access to emergency and surgical care in sub-Saharan Africa: The infrastructure gap. *Health Policy Plan* 2012;27(3):234-44.

22. Deckelbaum DL, Ntakiyiruta G, Liberman AS, Razek T, Kyamanywa P. Augmenting surgical capacity in resource-limited settings. *The Lancet* 2011;.

23. Funk LM, Weiser TG, Berry WR, Lipsitz SR, Merry AF, Enright AC, et al. Global operating theatre distribution and pulse oximetry supply: An estimation from reported data. *The Lancet* 2010;376(9746):1055-61.

24. De Vries EN, Prins HA, Crolla RMPH, Den Outer AJ, Van Andel G, Van Helden SH, et al. Effect of a comprehensive surgical safety system on patient outcomes. *N Engl J Med* 2010;363(20):1928-37.

25. Whyte S, Lingard L, Espin S, Baker GR, Bohnen BA, Orser B, et al. Paradoxical effects of interprofessional briefings on OR team performance. *Cognition, Technology and Work* 2008;10(4):287-94.

For peer review only

For peer review only - http://bmjopen.bmj.com/site/about/guidelines.xhtml

26. Wilson D. Who Guards the Guardians? Ian Kennedy, Bioethics and the 'Ideology of Accountability' in British Medicine. *Social History of Medicine* 2011; July 01;.

27. Aiken AM, Karuri DM, Wanyoro AK, Macleod J. Interventional studies for preventing surgical site infections in sub-Saharan Africa - A systematic review. *International Journal of Surgery* 2012;10(5):242-9.

28. Siddiqi K, Newell J, Robinson M. Getting evidence into practice: What works in developing countries?. *International Journal of Quality in Health Care* 2005;17:447-54.

29. Edmondson AC. Speaking up in the operating room: How team leaders promote learning in interdisciplinary action teams. *Journal of Management Studies* 2003;40(6):1419-52.

30. McCulloch P, Rathbone J, Catchpole K. Interventions to improve teamwork and communications among healthcare staff. *British Journal of Surgery* 2011;98(4):469-79.

31. Ider B-, Adams J, Morton A, Whitby M, Muugolog T, Lundeg G, et al. Using a checklist to identify barriers to compliance with evidence-based guidelines for central line management: A mixed methods study in Mongolia. *International Journal of Infectious Diseases* 2012;16(7):e551-7.

32. Kotagal M, Lee P, Habiyakare C, Dusabe R, Kanama P, Epino HM, et al. Improving quality in resource poor settings: Observational study from rural Rwanda. *BMJ* 2009;339:1311-3.

33. Walker IA, Merry AF, Wilson IH, McHugh GA, O'Sullivan E, Thoms GM, et al. Global oximetry: An international anaesthesia quality improvement project. *Anaesthesia* 2009;64(10):1051-60.

For peer review only

For peer review only - http://bmjopen.bmj.com/site/about/guidelines.xhtml

A qualitative study comparing experiences of the surgical safety checklist in hospitals in high- and low-income countries

Journal:	*BMJ Open*
Manuscript ID:	bmjopen-2013-003039.R1
Article Type:	Research
Date Submitted by the Author:	05-Jul-2013
Complete List of Authors:	Aveling, Emma-Louise; University of Leicester, Health Sciences<br>McCulloch, Peter; University of Oxford, Nuffield Surgery<br>DixonWoods, Mary; University of Leicester, Health Sciences
**Primary Subject Heading**:	Surgery
Secondary Subject Heading:	Qualitative research, Health services research
Keywords:	HEALTH SERVICES ADMINISTRATION & MANAGEMENT, International health services < HEALTH SERVICES ADMINISTRATION & MANAGEMENT, Protocols & guidelines < HEALTH SERVICES ADMINISTRATION & MANAGEMENT, Quality in health care < HEALTH SERVICES ADMINISTRATION & MANAGEMENT, QUALITATIVE RESEARCH, SURGERY

SCHOLARONE™
Manuscripts

A qualitative study comparing experiences of the surgical safety checklist in hospitals in high- and low-income countries

Emma-Louise Aveling,[1*] Peter McCulloch,[2] Mary Dixon-Woods [3]

[1] Research Associate, Department of Health Sciences, University of Leicester, 22-28 Princess Road West, Leicester LE1 6TP

[2] Clinical Reader in Surgery, Nuffield Department of Surgical Science, University of Oxford

[3]Professor of Medical Sociology, Department of Health Sciences, University of Leicester, 22-28 Princess Road West, Leicester LE1 6TP

*Corresponding author: Emma-Louise Aveling, Department of Health Sciences, University of Leicester, 22-28 Princess Road West, Leicester LE1 6TP, UK

Email: eea5@le.ac.uk; Tel: +44116 252 3287

For peer review only

For peer review only - http://bmjopen.bmj.com/site/about/guidelines.xhtml

Article summary

ARTICLE FOCUS

- Bold claims have been made for the ability of the WHO surgical checklist to reduce surgical morbidity and mortality and improve patient safety.
- We aimed to identify and compare influences on checklist implementation and compliance in operating theatres in two UK hospitals and one African hospital.

KEY MESSAGES

- Consistent use, completion and fidelity of checklist deployment are not straightforward in any setting, but may be higher in the two UK hospitals compared with the hospital in a low-income country.
- Contrary to claims in early studies of the checklist, additional resources and changes to clinical systems may be needed to secure compliance with the checklist and its principles in low-income contexts.
- The checklist is no magic bullet; improvements in communication and quality of team interactions do not automatically follow its introduction in any setting, and the checklist may indeed introduce new, unintended risks in low-income settings.

STRENGTHS AND LIMITATIONS

- Strengths: detailed, first-hand observations of the implementation of the surgical checklist in diverse settings; identification of key lessons of practical value in securing the benefits of the checklist and avoiding unintended consequences, especially in low-income countries.
- Limitations: data on outcomes were unavailable; observations conducted at different stages of implementation; some evidence of observer effect; limited number of sites; details of training were unreliable.

For peer review only - http://bmjopen.bmj.com/site/about/guidelines.xhtml

ABSTRACT

Objective: Bold claims have been made for the ability of the WHO surgical checklist to reduce surgical morbidity and mortality and improve patient safety regardless of setting. Little is known about how far the challenges faced by low income countries are the same or different as those in high income countries. We aimed to identify and compare influences on checklist implementation and compliance in the UK and Africa.

Design: Ethnographic study involving observations, interviews, and collection of documents. Thematic analysis of data.

Setting: Operating theatres in one African university hospital and two UK university hospitals.

Participants: 112 hours of observations were undertaken. Interviews with 39 theatre and administrative staff were conducted.

Results: Many staff saw value in the checklist in the UK and African hospitals. Some resentment was present in all settings, linked to conflicts between the philosophy behind the checklist and the realities of local cultural, social and economic contexts. Compliance – involving use, completeness and fidelity - was considerably higher, though not perfect, in the UK settings. In these hospitals, compliance was supported by established structures and systems, and was not significantly undermined by major resource constraints; the same was not true of the low-income context. Hierarchical relationships were a major barrier to implementation in all settings, but were more marked in the low-income setting. Introducing a checklist in a professional environment characterised by lack of accountability and transparency could make staff feel jeopardised legally, professionally, and personally, and it encouraged them to make misleading records of what had actually been done.

Conclusion: Surgical checklist implementation is likely to be optimised, regardless of setting, when used as one tool in multi-faceted cultural and organisational programmes to strengthen patient safety. It cannot be assumed that the introduction of a checklist will automatically lead to improved communication and clinical processes.

For peer review only

For peer review only - http://bmjopen.bmj.com/site/about/guidelines.xhtml

INTRODUCTION

Surgery provides immediate, transformative treatment for many conditions, usually in a single episode, and will remain a key therapeutic strategy in all countries. Surgery is, unfortunately, also a major source of avoidable morbidity and mortality worldwide,[1, 2] though substantial improvements can be achieved by reducing variation in the reliability of surgical care processes.[3] Checklists are increasingly promoted as a way to deliver such reliability, following the pilot study of the World Health Organization's (WHO) Surgical Safety Checklist (Box 1) which reported reductions in mortality and complications in surgical patients.[4] The checklist involves 19 separate items to be checked at three distinct points: the Sign In, before induction of anaesthesia; the Time Out, before skin incision; and the Sign Out, at the end of the procedure.

One important and unusual feature of the WHO checklist is its claim to universality. The checklist pilot study[4] was distinctive for its being conducted in high, middle and lower income settings, including one African hospital. It concluded that the checklist programme could "improve the safety of surgical patients in diverse clinical and economic environments" (p496). It also suggested that implementation was neither costly or lengthy, proposing that only two items – pulse oximetry and prophylactic antibiotics - would involve commitment of significant resources; both were reported to be available at all sites in the study (including the low-income country hospitals), though used inconsistently.

A second important feature of the WHO checklist is that it combines checks for technical items (such as administration of antibiotics and use of pulse oximeters) with other so-called "non-technical" items (such as team introductions and confirmations of procedures) whose principal purpose is to promote aspects of teamwork, communication and situational awareness. Inclusion of the non-technical items in the checklist was influenced by research demonstrating an association between team practices (e.g. communication behaviours) and improved safety processes and attitudes.[5, 6] Performing the checklist is also envisioned as a non-technical intervention in its own right: the checks are to be performed orally, and are, according to the checklist pilot study, "intentionally designed to create a collective awareness among surgical teams of whether safety processes are being completed" (p497).[4]

The potential for surgical safety checklists to improve safety and outcomes and generate substantial cost savings[7] has attracted global interest. In part owing to the WHO's Safe Surgery Saves Lives campaign, around 1800 institutions are now reported to be using the checklist worldwide.[8] Studies in high- and low-income settings have continued to report positive impacts on surgical outcomes, but the size of the reported effects varies across and within settings, possibly because of variability in compliance.[9-13] Compliance can be heuristically distinguished into three dimensions: use, completeness and fidelity. Use refers to whether the checklist is used at all; completeness the extent to which it is completed in full, without items being skipped; and fidelity the extent to which items are performed as intended, with items ticked as complete only when checks have genuinely been made, at the right time, and in communication with the whole team.

The checklist pilot study[4] and subsequent studies of resource-poor settings[9] were focused on assessment of outcomes, and did not characterise the implementation processes and characteristics of contexts likely to optimise checklist compliance. Yet implementing checklists, securing compliance and replicating positive outcomes necessarily require an appreciation of socio-cultural context.[14] Healthcare practitioners do not blindly follow procedures; rather, compliance often depends on workers' perceptions of effectiveness, relevance and rationale, their efforts to juggle competing professional, moral and social norms and demands, and the institutional and socio-economic contexts

For peer review only - http://bmjopen.bmj.com/site/about/guidelines.xhtml

in which they work.[15, 16] Studies of checklist implementation in high-income countries suggest that challenges to compliance are linked to organisational safety culture,[14] and may include hierarchical structures, dismissive attitudes, absence of key team members, and hurrying through checks.[17-19] Yet study of the checklist in low-income countries has been largely neglected. This is an important omission: complications and mortality following surgery in sub-Saharan Africa remain far higher than in developed countries[1] and almost 200 registered institutions in Africa are now reported to be using the checklist.[20] The specific challenges likely to be faced in such settings remain under-researched. We aimed to identify and compare influences on checklist implementation and compliance in operating theatres in hospitals in high-income and low-income countries.

METHODS

Our study design involved ethnographic case studies[21] in three hospitals: two in the UK (anonymised as Amfield and Tolgrave) and one (anonymised as Mbile) in a low-income sub-Saharan African country. The UK centres were two large university hospitals in major urban settings that were participating in a larger research project on culture and behaviour relating to patient safety. The African centre was a university hospital serving a mixed urban and rural population of approximately 4-5 million. All three hospitals had adopted, as official policy, a checklist based on the WHO template. The checklist had been mandated for use in all UK hospitals by the National Patient Safety Agency two years before our study; in Mbile, it was introduced one year before as part of an externally funded international collaboration to improve surgical safety. Given the sensitivity of some findings, we do not provide further details that could make participating sites or individual staff identifiable. Ethical approvals were obtained for the study in the UK and in the African site.

Data collected across all three sites comprised: i) semi-structured interviews with anaesthetists, surgeons, theatre staff, management and administrative staff, and covered experiences of checklist implementation, institutional context, and management of patient safety. Interviews were conducted with informed consent from participants and were recorded, translated (where required) and transcribed; ii) non-standardised observations and informal discussions with staff in operating rooms, recorded as field-notes in notebooks and later elaborated more fully; iii) collation of relevant documentation. Observations were conducted in all four operating rooms in the African site. In the UK sites, observations were undertaken in a subset of theatres that covered a diversity of surgical specialities (including general, orthopaedic, oral-maxillofacial and vascular surgery). Interviewees were recruited after a period of observations had been completed, allowing the researchers to familiarise themselves with the setting and the organisation of theatres and theatre teams. Recruitment was guided by purposive sampling so that the sample was diverse in terms of seniority of staff (trainees to consultants and theatre managers), sub-specialty and disciplines (see Table 1). No one approached declined to be interviewed (although one staff member declined to have her interview recorded on religious grounds). ELA collected the data in two sites; a second observer (see acknowledgements) collected data in one of the UK sites. ELA analysed the data from all three sites. ELA and MDW independently reviewed a sample of data and together came to agreement on interpretation.

Data were analysed thematically,[22] supported by Nvivo software, and guided but not constrained by sensitising concepts derived from the research questions.[23] A single coding framework was iteratively developed, refined and applied to all three data sets. Data were initially coded into basic-themes within three global categories - implementation barriers, facilitators and contextual characteristics. In each category, basic codes were grouped into organising themes, and patterns in different sites were identified, compared and contrasted, paying attention to interrelations between themes. In addition, we analysed data from each site to identify patterns of use, completeness and fidelity of checklist compliance.

For peer review only - http://bmjopen.bmj.com/site/about/guidelines.xhtml

RESULTS

We interviewed 39 staff: 19 in Mbile and 20 across the two UK hospitals (see Table 1). A total of 112 hours of observations were undertaken: 60 hours over two weeks in Mbile, 28 hours over four days in Amfield and 24 hours over three days in Tolgrave.

< TABLE 1 ABOUT HERE>

In all three sites, checklist implementation strategies included staff training, in situ demonstrations and awareness-raising at departmental meetings. In Mbile, training was assisted by two different international organisations, but surgeons were trained separately from other staff. This resulted in some mixed messages being circulated within the operating department.

Though in all three hospitals some staff attributed initial problems with checklist compliance to lack of awareness or understanding, most staff in both the UK and African hospitals could describe broadly how it was *intended* to be used. In all hospitals, many staff saw considerable value in the checklist. UK staff tended to emphasise prevention of rare but potentially catastrophic errors, such as wrong site surgery, and also suggested checklist use could improve communication and teamwork with "no hierarchy" (Amfield, anaesthetist).

> *We're trying to prevent what are usually rare errors, rare mistakes, you know, the majority of the things on that checklist are done most of the time without the checklist, but every now and then [...] you forget to check if you're operating on the right leg and not the left leg, and that's rare, but on very rare occasions it then leads to a disaster. [Amfield, Anaesthetist, U021]*

> *It's not something fluffy and friendly, it is actually functional and it is about all respecting one another [Tolgrave, Anaesthetist, U014]*

Mbile staff reported that few other protocols or standardised checks were in place in their hospital; some therefore welcomed the prompting and structure provided by the checklist. In contrast to the UK sites, they emphasised the checklist's value in catching more common mistakes – such as forgetting prophylactic antibiotics – but did not mention a role for the checklist in undermining hierarchies or improving teamwork.

> *Whenever we do the checklist we identify that some things have been missed [Mbile, Anaesthetist, A005]*

Compliance

Nurses or theatre practitioners in all three hospitals had primary responsibility for initiating use of each checklist section, but differences in the way the checks were performed were evident. In UK hospitals, staff performed checks out loud in front of all team members who were present. In Mbile, checks were undertaken by an individual nurse, who ticked boxes based on his/her perceptions of what had happened or quietly checked on a one-to-one basis whether designated individuals had remembered specific tasks.

> *[During the procedure] I see the nurse filling in the checklist, which he then puts on top of the cupboard. He doesn't speak to anyone in the process, except to talk quietly with one of the surgical residents. [Mbile fieldnotes]*

Though our study was not designed as a systematic audit, interviews and observations suggested that checklist use, completion, and fidelity, while not perfect, were more consistent in UK settings compared with Mbile. In the UK hospitals, the checklist was *used* in all procedures that we observed, documentation was fully *completed*, and interviewees reported internal audits showing checklist use

For peer review only - http://bmjopen.bmj.com/site/about/guidelines.xhtml

in over 90% of procedures. *Fidelity* was more variable: in some instances teamwork was undermined by staff being distracted, dismissive, or absent during checks. During Time Out, staff often continued with other preparatory tasks, meaning it was not always the “moment of silence it’s supposed to be” (nurse, Tolgrave). Sign In, supposed to be done before induction of anaesthesia, was often performed at the same time as the Time Out when the patient was already anaesthetised. Sign Out was often rushed or cursorily performed, though equipment counts were extremely thorough.

> *The scrub nurse and the student Operating Department Practitioner (ODP) are sort of removed, standing over by their metal trolley preparing some of the equipment and talking; they’re not really listening to the rest of the group [surgeon, anaesthetist, ODP] who are crowded around patient (who’s already anaesthetised), but they answer the questions when they’re called to, and they do all the checks. [Amfield fieldnotes]*

> *At the end of the operation, just as the patient is being moved from the operating table onto a trolley, and they’re waiting for him to wake up [..] the anaesthetist says “oh, we haven’t done the sign out, oh, we should do the sign out”. By this point the surgeon’s already left the theatre [Amfield fieldnotes]*

In Mbile, *use* of the checklist was highly inconsistent: participants’ estimates ranged from ‘always’ to ‘hardly ever’, and direct observations induced a Hawthorne effect, where checklist use increased from few procedures early in our observation period to all procedures by the final two days. When staff were under pressure due to staff shortages, emergencies, or lengths of shifts (up to 36 hours) not seen in the UK, the checklist was apt to be abandoned altogether.

> *Sometimes it’s difficult to use this [checklist], due to staff overload, so sometimes if there is an emergency case, they may not fill it in. Rather than fill it in, they might just get the instruments for those guys because of the urgency of the case [Mbile, Theatre nurse, A011]*

Completeness was both reported and observed to be highly variable in Mbile, with no improvement over the period of observations. *Fidelity* was also problematic in Mbile. Check-boxes were often ticked without the requisite information having been obtained or the tasks to which they referred undertaken, and the timing of checks was haphazard. Sometimes the nurse ticked the Sign-In and Time-Out checkboxes when the procedure was already underway, rendering them useless. Team introductions were never performed, yet the box was always ticked. Sometimes the Sign-Out boxes were ticked before the procedure was completed. This meant that equipment counts were ticked as complete when equipment was still in use, and specimens were recorded as correctly labelled before they had been removed from the patient. If equipment counts were performed at all in Mbile, they tended to be unreliable, completed in silence (not out loud as specified by the WHO guidance) by a single nurse at variable points during the procedure using a form that did not list all relevant equipment.

> *[During the procedure] I check the checklist: the nurse had (at some point before the end of the operation) filled the entire thing in, including “specimen is correctly labelled” – even though the specimen-to-be was still inside the patient. [Mbile fieldnotes]*

> *The nurse fills in the back side of the form (the equipment count chart), even though all the equipment used in the operation has been cleared away, and completes the sign-out column of the checklist, without talking to anyone. [Mbile fieldnotes]*

Factors influencing checklist implementation

Some reasons for variable compliance were similar between the UK and African sites; others were different, and some problems that appeared to be the same had different underlying mechanisms. A shared barrier to consistent compliance across the UK and African settings was a perception among

For peer review only - http://bmjopen.bmj.com/site/about/guidelines.xhtml

some that the checklist was futile, or that some checks (particularly the non-technical ones) were a waste of time.

> *The anaesthetist refuses to say his name. He says "I think we all know each other by now [...] it's ridiculous" [Amfield fieldnotes]*

> *No, no, no [we don't do introductions] We know each other! I know the anaesthetist, I know the nurse. [Mbile, Surgeon, A017]*

Perceptions that some elements of the checklist were pointlessness were partly based on the (demonstrably false) view among some staff at all three hospitals that incidents the checklist was designed to prevent would never happen in their environments. In all hospitals the persuasive power of sentinel events – even very serious incidents (including, in Mbile, major operations on the wrong patients and a wrong site surgery in Amfield) – was variable.

> *I can clearly remember two occasions where the surgeon was just very dismissive and it was like, 'oh I'm not doing this, I don't need to do this, I'm not' you know... and literally just walked away. [Amfield, Operating Department Practitioner, U020]*

> *Even though training on the checklist was given for surgeons, they don't use it, they don't believe in this bit of paper, because mostly they said, 'we don't mistake the identity of the patient, it doesn't happen that we get the wrong patient' [Mbile, Theatre nurse, A012]*

Differences in factors influencing compliance, and their underlying mechanisms, derived from the very different contexts of implementation. Perhaps the most obvious difference between the settings concerned material resources. In all settings, the technical items were broadly accepted as established good practice. In the UK hospitals, equipment and medicines were available to address these items.

Mbile's infrastructure was very different. The electricity supply, for example, was unreliable; even basic equipment, such as gauze, was not reliably available. Such resource shortages meant that it was not always possible to comply with the technical items on the checklist, yet nurses often ticked the boxes nonetheless. For example, there were no surgical markers at Mbile hospital, but documentary analysis showed the question 'is the site marked?' was always ticked 'yes'. The box for prophylactic antibiotics was also among those most consistently completed ('yes' or 'not applicable' were usually ticked), but, with only a limited range of antibiotics available and no hospital policy regarding administration of prophylactic antibiotics, doctors sometimes delayed antibiotic use in case of subsequent post-operative infections. There were too few pulse oximeters available for the number of operations being performed. Anaesthetists were fully aware of the value of pulse oximeters, but were often unable to access one; absence of the device rarely led to cancellation of an operation.

> *We don't have too many types of antibiotics [...] if we have given [the patient] prophylactic antibiotics and organisms have already developed some resistance to the antibiotics, how are we going to treat this guy if he develops some type of infection later on? That is the problem [...] but we really don't have single view in this issue, I mean, some people give antibiotics, some people don't give, it's not a standardised policy [Mbile, Surgeon, A019]*

> *In set-ups like ours, for example if there is no pulse oximetry, [..] if you consider it as a must we're going to lose many people because often we don't have this pulse oximetry. So if you say now I'm not going to anaesthetise a patient because I have no pulse oximeter definitely we will lose many patients that could have been helped if you operate on them without pulse oximeter. [Mbile, Anaesthetist, A002]*

For peer review only - http://bmjopen.bmj.com/site/about/guidelines.xhtml

The non-technical items on the checklist were far less dependent on material resources, yet they were much more likely to attract criticism and scepticism from staff. Some staff in all hospitals (UK and African) objected to the performance of the checklist in its entirety; more often, specific items were the subject of complaint. In the UK sites, a recurring objection was that the checklist duplicated pre-existing systems and checks, including, for example, long-standing equipment counting procedures. Perceptions of “duplicative” or “useless” checks contributed to the view of some in all settings that the checklist was an illegitimate bureaucratic intrusion, and resentment towards it intensified during busy periods, emergencies or staff shortages. The most deeply felt and repeatedly vocalised objections came from surgeons. Given strongly hierarchical team dynamics in both the UK and African hospitals, surgeons’ behaviour and attitudes in relation to the checklist were highly influential. This made it difficult for others to directly challenge defiant or dismissive surgeons.

> *I think it’s difficult because there is a hierarchy within the theatre complex, or within the medical profession full stop, and the theatre staff might feel intimidated by some of the environments they are in. Some of them are forceful enough and have a voice, but others are quite timid and probably quite reluctant to actually speak up. [Tolgrave, Anaesthetist, U012]*

> *The surgeon may not volunteer to cooperate to fill that checklist. [...] The nurse may stop her work, or may do it without filling the checklist. It may depend on the confidence of the nurse. [Mbile, Theatre Nurse, A013]*

Despite these objections, use of the checklist was high in the UK hospitals. Staff in these hospitals had been highly sensitised to issues of patient safety through exposure to their institutions’ policies and, though they did not see all patient safety interventions as positive, they felt that they had “a microscope on us about patient safety” (Tolgrave, nurse). Audit and feedback of data on checklist use and relevant outcomes (e.g. infection rates) were used to support checklist implementation and were tied to sanctions, which could include disciplinary action against staff who refused to comply.

> *You know I do not mind being part of any audit at all because I think it’s great, we won’t find anything or improve anything unless we do. [Tolgrave, ODP, U001]*

> *We have to be careful because you know, it’s in our registration as well to follow this protocol isn’t it, so [even] if the surgeon is not keen to do it, we have to tell them, you have to stop and do it [Tolgrave, ODP, U 008]*

These UK audits were not, however, perfect instruments, not least because they generally failed to capture *how* checklists were being used. Beyond formal arrangements, some local leaders acted as champions for the checklist. Surgeons, anaesthetists and senior nurses in both UK hospitals took on this role, and were important in leading by example and supporting junior or non-surgical staff when there was “push-back”.

> *We are there to iron out any problems, or if anybody gives them grief we can go in and fire it back if needed. [Tolgrave, Site coordinator, 002]*

In Mbile, introducing the checklist involved much more far-reaching change than in the UK. The absence of an established tradition of equipment counts, for example, meant that the checklist was a much more disruptive and demanding intervention in the African setting. Items that were non-controversial in a UK setting, such as documenting patient consent, had different meaning in a context where patients often lacked literacy and surgery was considered a privilege. The surgical checklist was one of the first explicit patient safety interventions introduced in Mbile. Staff had little prior exposure to the ideas and principles of the science of safety, and systems to support patient safety were poorly developed. The hospital lacked an established culture of audit; there was little

For peer review only - http://bmjopen.bmj.com/site/about/guidelines.xhtml

routine collection of clinical data and staff had no clear sense of the impact of the checklist on patient safety. There was no monitoring of checklist use, and no consequences for non-compliance.

> *I asked every department [if they want to do an audit] and they have not volunteered […] I think they're busy. It is not familiar, clinical audit. Most health workers don't know what clinical audit means. [Mbile, Administrator, A001]*

> *We really don't have the real number [for infection rates], how many patients have been affected, because we haven't applied those measurements, we really don't know [Mbile, Surgeon, A020]*

> *It's ok, nothing happens. For those who don't fill in the checklist there is no problem, no consequence. [Mbile, Theatre nurse, A011]*

Team dynamics were especially challenging in Mbile. Though local championing of the checklist was not absent, it was not highly visible either. Surgeons were the only doctors in the room: anaesthetists were not physicians, but held a BSc in anaesthesia. In observations, challenge over checklist use was very rarely witnessed. Wider societal hierarchies and cultural norms, including those relating to age, gender and education, resulted in particularly steep authority gradients. Nurses were often female and younger than the (exclusively) male surgeons, and had been socialised to be deferential and submissive. The working environment was described as lacking in transparency and accountability, particularly in relation to staff dismissals or promotions.

> *There were complaints that there is impartial [unfair] treatment between people. This is one of the problems from the managerial aspect. [Mbile, Anaesthetist, A002]*

The external socio-legal context in Mbile was one that staff felt was at best unreliable. At worst it was seen corrupt and unjust, offering little protection to vulnerable, low-status individuals. Interviews suggested that challenging those in authority could therefore feel very risky and have unpredictable results, perhaps damaging career prospects or even worse (Box 2). The checklist thus introduced new, unintended risks for staff.

<Box 2 about here>

DISCUSSION

The WHO's implementation manual identifies two purposes for the surgical checklist: ensuring consistency in patient safety and introducing (or maintaining) a culture that values achieving it.[24] Our study suggests that hospitals in both high and low-income countries may experience challenges in delivering on these aims, even when many staff (non-physicians in particular) can see value in the checklist. An important finding is the extent to which hospitals in both low and high-income countries encountered the same obstacles in implementing the checklist; the key differences related to contexts. Material contexts were perhaps most vividly distinct: resource shortages meant that it was often impossible to comply with the checklist's technical requirements in Mbile. Resource and infrastructure constraints in low-income countries have been well-described previously,[25-29] suggesting that the problems in our African study hospital were unlikely to be unique. Kwok and colleagues,[9] for example, similarly identified insufficient numbers of pulse oximeters as a barrier to checklist compliance, and suggest that the hospitals in the original pilot study were perhaps not representative of those in low-income settings that lack basic resources. Greater acknowledgement may therefore be needed that additional material resources may well be required to implement the checklist in low-come countries. Many of the non-technical items on the checklist do not, of course, require additional material resources, but rather changes in communication practices and teamwork. Hierarchical team dynamics undermining of safety were evident in all three hospitals in our study, but were more pronounced in the African setting. The assumption that improvements in communication and team

For peer review only - http://bmjopen.bmj.com/site/about/guidelines.xhtml

For peer review only

interactions automatically follow the introduction of a checklist[4, 30] is thus open to question. Our study points to a much more complex interrelation between checklist procedures, context, 'culture' and behavioural changes, and confirms that checklists should not be regarded as magic bullets.

Contextual differences meant that Mbile had much more to do to implement and secure compliance with the checklist, and it lacked features that supported implementation in the UK sites. Local policies, institutional focus and support, well-established audit and data collection systems, clear lines of accountability with consequences for staff and hospitals for non-compliance were all more pronounced (if not always perfect) in the UK hospitals than their African counterpart. They were important indirectly, by signalling the institutional priority given to patient safety,[18] and directly (e.g. disciplinary action). Despite these advantages, the UK sites also, showed evidence of persisting problems of fidelity, audits that focused too much on documentation of box-ticking, and resentment of perceived top-down initiatives.

Our study produced some disquieting evidence that poor checklist implementation in low-income settings might not only fail to reduce patient safety risks, but also *introduce* new risks for staff and/or patients. Previous work has also shown that even in well-resourced settings, checklists may not consistently deliver positive effects, and may potentially produce some paradoxical or harmful effects including inhibition of team processes.[5, 31] These may be even more consequential in a low-income setting. The style of checks in Mbile limited improvements in team communication, and was directly contrary to the design intent of creating collective awareness. The authoritarian status of the surgeon elite contributed to these unhelpful features of team dynamics, which were exacerbated by wider societal hierarchies and lack of transparency in institutional processes. Though unhelpful team dynamics were also sometimes evident in the UK hospitals, introducing a checklist in a professional environment characterised by a lack of accountability and transparency raised additional ethical concerns in Mbile: it made some staff feel jeopardised legally, professionally, and personally, and it encouraged them to make misleading records of what had actually been done. Thus, although many staff welcomed attempts to introduce structure and standardisation to procedure the preparation and organisation of procedures, the benefits for patient safety were unclear, especially as no audit data were available to establish outcomes.

None of this is to deny the potential of the checklist to deliver significant benefits for the safety of patients in low-income settings, but it is to emphasise the need for a high level of sensitivity to cultural and economic contexts in the design and implementation of interventions. Introduction of the checklist may not, of itself, address underlying deficits in the clinical processes the checks cover. Workers cannot comply with checklist items that require material resources (e.g. antibiotics and pulse oximeters) that are not available in their organisations. Many non-technical items, of course, represent potentially low-cost ways to improve care and may thus be attractive for resource-constrained environments, particularly when many healthcare interventions are so expensive. But attempts to change the status quo in these settings should be informed by a sound evidence base, attention to unintended consequences and recognition of the influence of local histories.[32] Though the volume of research on patient safety in low-income countries is now increasing,[33, 34] little is known about which kinds of strategies are most effective in addressing patient safety issues in these settings.[35]

Our study suggests that explicit interventional focus on improving team dynamics and communication may be even more critical in African healthcare settings than high-income countries. Professional groups – in whatever setting - should be trained together on the checklist, not separately as occurred in Mbile. Additional interventional focus on relevant clinical systems may also be necessary. For example, our findings suggest that where equipment count practices are not well established, focused training, agreed procedures and on-going support to implement these practices are needed if the use of checklists is to be meaningful. Our findings also reinforce lessons from improvement science more broadly concerning the importance of collection and feedback of data; in low-income settings where

For peer review only - http://bmjopen.bmj.com/site/about/guidelines.xhtml

audit is not such an established feature of institutional governance, additional capacity building may be needed. [36]

The critical role of local leads in motivating the adoption of new ideas[37] means that particular efforts must be made to secure buy-in from surgeons and to ensure that junior or non-medical staff are assured of institutional support. Multiple, senior champions from across the relevant disciplines is needed to ensure leadership is visible and persuasive. Another effective tactic may be to empower theatre staff to call on supportive senior surgeons ('champions') for back-up when they face 'push back' from other staff.[38] While high-level institutional strategies were seen by some as misdirected or problematic in UK settings, the comparison with Mbile nonetheless underscores the value of all front-line staff having support and clear leadership for their efforts to improve safety. Such institutional support should also include the judicious use of 'hard edges'[38] and mechanisms through which there are predictable consequences for staff – at all levels – when patient safety is jeopardised. As patient safety becomes more firmly established on local and national health agendas in low-income countries, for example through initiatives such as the WHO's Global Patient Safety Challenge,[39] institutional and infrastructural support for safety is likely to improve as well. Attention to strategies likely to improve implementation (Box 3) may help with this.

Our study has a number of limitations. It did not include data on outcomes. Mbile had only been using the checklist for one year, in contrast to two years in UK hospitals, so was at a different stage of implementation. Despite the importance of training to secure checklist compliance,[40] the precise nature of what was provided in the three sites (especially in Mbile) was unclear, due to unreliable (and in some cases conflicting) accounts of events. A clear observer effect was found in Mbile, and may also have occurred in UK hospitals. Observations over a longer period in all sites might have identified more influences on checklist compliance. Nonetheless, our study has yielded important lessons for implementing surgical checklists in different settings.

Our work demonstrates that assumptions about context are encoded into patient safety tools. Optimising checklist deployment requires careful analysis of these assumptions, and of the extent to which they match local set-ups or require the introduction of new systems and practices. It also demonstrates that changes in teamwork practices do not automatically follow checklist introduction, even where non-technical items are included, but rather require explicit, interventional focus. Checklists are neither a 'quick-fix' nor a tool that can be effectively implemented in isolation; in resource-constrained settings, they are especially unlikely to be free of costs and risks. Safety checklists are most likely to be effective and sustainable when implemented as part of broader, multi-faceted programs addressing social, behavioural, logistical and organisational issues,[14, 26, 41, 42] where there is strong institutional focus on patient safety, multi-disciplinary leadership, monitoring systems in place, and consequences at all levels for non-compliance.

<Box 3 about here>

For peer review only - http://bmjopen.bmj.com/site/about/guidelines.xhtml

**Role/ Profession**	**Mbile (African hospital)**	**Amfield and Tolgrave (UK hospitals)**
Anaesthetists	4	4
Surgeons	6	3
Managers/administrators	4	4
Nurses and Theatre Practitioners	5	9
Total	***19***	***20***

Table 1. Number of individual interviews by profession in African and UK sites

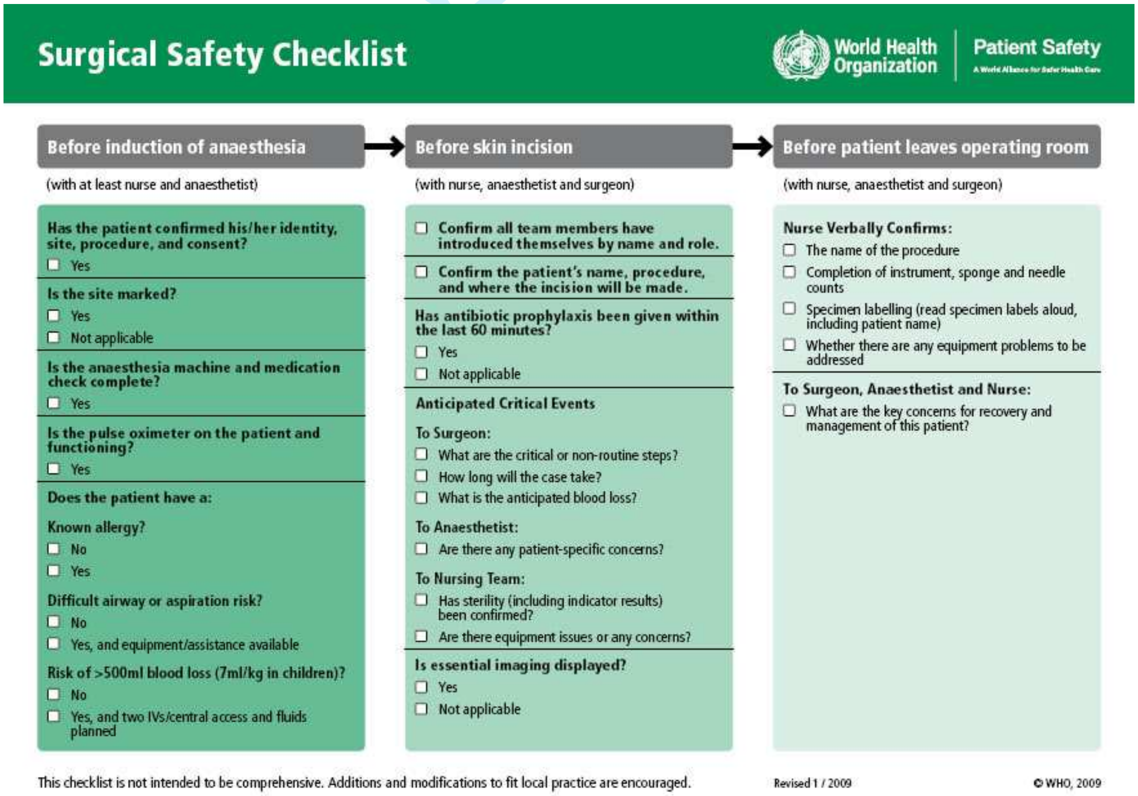
Surgical Safety Checklist

World Health Organization | Patient Safety

Before induction of anaesthesia

(with at least nurse and anaesthetist)

Has the patient confirmed his/her identity, site, procedure, and consent?
- ☐ Yes

Is the site marked?
- ☐ Yes
- ☐ Not applicable

Is the anaesthesia machine and medication check complete?
- ☐ Yes

Is the pulse oximeter on the patient and functioning?
- ☐ Yes

Does the patient have a:

Known allergy?
- ☐ No
- ☐ Yes

Difficult airway or aspiration risk?
- ☐ No
- ☐ Yes, and equipment/assistance available

Risk of >500ml blood loss (7ml/kg in children)?
- ☐ No
- ☐ Yes, and two IVs/central access and fluids planned

Before skin incision

(with nurse, anaesthetist and surgeon)

- ☐ **Confirm all team members have introduced themselves by name and role.**
- ☐ **Confirm the patient's name, procedure, and where the incision will be made.**

Has antibiotic prophylaxis been given within the last 60 minutes?
- ☐ Yes
- ☐ Not applicable

Anticipated Critical Events

To Surgeon:
- ☐ What are the critical or non-routine steps?
- ☐ How long will the case take?
- ☐ What is the anticipated blood loss?

To Anaesthetist:
- ☐ Are there any patient-specific concerns?

To Nursing Team:
- ☐ Has sterility (including indicator results) been confirmed?
- ☐ Are there equipment issues or any concerns?

Is essential imaging displayed?
- ☐ Yes
- ☐ Not applicable

Before patient leaves operating room

(with nurse, anaesthetist and surgeon)

Nurse Verbally Confirms:
- ☐ The name of the procedure
- ☐ Completion of instrument, sponge and needle counts
- ☐ Specimen labelling (read specimen labels aloud, including patient name)
- ☐ Whether there are any equipment problems to be addressed

To Surgeon, Anaesthetist and Nurse:
- ☐ What are the key concerns for recovery and management of this patient?

This checklist is not intended to be comprehensive. Additions and modifications to fit local practice are encouraged.

Revised 1 / 2009 © WHO, 2009

Box 1: WHO Surgical Safety Checklist (reproduced with the permission of the WHO; available from http://www.who.int/patientsafety/safesurgery/tools_resources/SSSL_Checklist_finalJun08.pdf)

For peer review only - http://bmjopen.bmj.com/site/about/guidelines.xhtml

A patient admitted for cholecystectomy suffered hypoxic brain injury and died following surgery. Subsequently, two staff members (not the surgeon) were threatened with guns by the patient's family, who said the surgical team had killed the patient. The two staff members were later arrested and criminal charges brought against one of them. One of the questions asked during the police investigation was whether a pulse oximeter had been used. It had not: according to staff, no pulse oximeter was available for use, even though the checklist requiring use of this equipment was, officially, in use at the hospital. Adoption of the checklist by the hospital, without adequate provision of the resources necessary for compliance, had left low-status front-line staff more socially and legally vulnerable, as it could be argued that they had failed to comply with hospital policy. Moreover, at all levels of the institution there was a great deal of uncertainty about staff rights and their employers' responsibilities in such circumstances. The accused staff were not provided with any legal representation for some weeks.

Box. 2: Description of events following an adverse event in Mbile, illustrative of the wider socio-legal context.

For peer review only - http://bmjopen.bmj.com/site/about/guidelines.xhtml

- Checklist implementation will be optimised when part of a broader, multi-faceted cultural and organisational programme to strengthen patient safety
- Introduction of the checklist does not automatically lead to changes in teamwork and communication, despite the inclusion of non-technical items. Hierarchical dynamics within the operating theatre may be particularly prominent in some low-income contexts, exacerbated by the wider socio-legal context
  - Improvements in communication and teamwork require direct interventional focus
  - Whole team training (rather than separate training for different professional groups) is important
- Assumptions about implementation contexts (e.g. resources, policies, systems in place) encoded within the checklist require careful scrutiny
  - Performance of the checklist may not be the only change(s) required
  - In resource-constrained settings especially, existing systems and resources may need to be strengthened for the checklist to be used with completeness and fidelity (e.g. provision of pulse oximetry)
- Local leadership and championing of the checklist is important and needs to:
  - Include support from the highest institutional level
  - Include senior staff, particularly senior surgeons
  - Include leaders from all professional groups
  - Be visible in theatres
- Monitoring (including process and impact measures) must be in place and fed back to all staff
- Poor compliance (use, completeness and/or fidelity) may not only fail to strengthen surgical safety - it may also introduce risks for staff and/or patients in some environments

Box 3. Lessons for checklist implementation in high- and low-income country settings

For peer review only - http://bmjopen.bmj.com/site/about/guidelines.xhtml

Declarations

Acknowledgements: Our sincere thanks to the staff of all three hospitals for their time and participation in the study. Our thanks to Joel Minion, who collected the data from one of the UK sites. We would also like to thank Dr Wynne Aveling for the clinical expertise he offered during data collection.

Ethics: Ethical approval was granted by the Leicestershire, Northamptonshire and Rutland Research Ethics Committee (number 10/H0406/77), and the Institutional Ethical Review Board for research in the African site. All participants gave informed consent.

Funding: We thank the Higher Education Innovation Fund Impact Award; Wellcome Trust Senior Investigator Award WT097899MA; and the Department of Health Policy Research Programme (reference number 0770017) for providing funding for this study.

Role of sponsor: The funders did not have a role in study design; in the collection, analysis, and interpretation of data; in the writing of the report or the decision to submit the article for publication. The research was conducted independently of the funders.

Contributorship: ELA and MDW jointly conceived and designed the study with support from PM. ELA negotiated access, recruited participants and conducted the fieldwork in one UK site and the African site. ELA analysed the data from all three sites. ELA and MDW conducted the literature review, with additional suggestions from PM. ELA and MDW led the writing of the manuscript. PM contributed clinical expertise. All authors contributed to interpretation of the findings, contributed to drafting the manuscript and approved the final version. ELA is the guarantor.

Data access: ELA had full access to all of the data in the study and can take responsibility for the integrity of the data and the accuracy of the data analysis. MDW had full access to the data from the UK sites and the opportunity to access the African data. For ethical reasons full data were not shared with PM.

Data sharing: No additional data available.

Competing interest declaration: All authors have completed the Unified Competing Interest form at www.icmje.org/coi_disclosure.pdf (available on request from the corresponding author) and declare that (1) ELA, MDW, PM] have no relationships with [any companies] that might have an interest in the submitted work in the previous 3 years; (2) their spouses, partners, or children have no financial relationships that (3) ELA, MDW, PM have no non-financial interests that may be relevant to the submitted work.

The Corresponding Author has the right to grant on behalf of all authors and does grant on behalf of all authors, a worldwide licence (http://www.bmj.com/sites/default/files/BMJ%20Author%20Licence%20March%202013.doc) to the Publishers and its licensees in perpetuity, in all forms, formats and media (whether known now or created in the future), to i) publish, reproduce, distribute, display and store the Contribution, ii) translate the Contribution into other languages, create adaptations, reprints, include within collections

For peer review only - http://bmjopen.bmj.com/site/about/guidelines.xhtml

and create summaries, extracts and/or, abstracts of the Contribution and convert or allow conversion into any format including without limitation audio, iii) create any other derivative work(s) based in whole or part on the on the Contribution, iv) to exploit all subsidiary rights to exploit all subsidiary rights that currently exist or as may exist in the future in the Contribution, v) the inclusion of electronic links from the Contribution to third party material where-ever it may be located; and, vi) licence any third party to do any or all of the above. All research articles will be made available on an Open Access basis (with authors being asked to pay an open access fee—see http://www.bmj.com/about-bmj/resources-authors/forms-policies-and-checklists/copyright-open-access-and-permission-reuse). The terms of such Open Access shall be governed by a Creative Commons licence—details as to which Creative Commons licence will apply to the research article are set out in our worldwide licence referred to above.

For peer review only

References

1. Bainbridge D, Martin J, Arango M, Cheng D, Evidence-based Peri-operative Clinical Outcomes Research (EPiCOR) Group. Perioperative and anaesthetic-related mortality in developed and developing countries: a systematic review and meta-analysis. *Lancet* 2012; Sep 22;380(9847):1075-81.

2. Anderson O, Davis R, Hanna GB, Vincent CA. Surgical adverse events: A systematic review. *Am J Surg* 2013; Apr 30;Epub ahead of print.

3. Kreckler S, Morgan RD, Catchpole K, New S, Handa A, Collins G, et al. Effective prevention of thromboembolic complications in emergency surgery patients using a quality improvement approach. *Bmj Quality & Safety* 2013; May 24;Epub ahead of print.

4. Haynes AB, Weiser TG, Berry WR, Lipsitz SR, Breizat AHS, Dellinger EP, et al. A surgical safety checklist to reduce morbidity and mortality in a global population. *N Engl J Med* 2009;260:491-9.

5. Lingard L, Regehr G, Orser B, Reznick R, Baker GR, Doran D, et al. Evaluation of a preoperative checklist and team briefing among surgeons, nurses, and anesthesiologists to reduce failures in communication. *Archives of Surgery* 2008;143(1):12-7.

6. Leonard M, Graham S, Bonacum D. The human factor: The critical importance of effective teamwork and communication in providing safe care. *Quality and Safety in Health Care* 2004;13(SUPPL. 1):i85-90.

7. Semel ME, Resch S, Haynes AB, Funk LM, Bader A, Berry WR, et al. Adopting a surgical safety checklist could save money and improve the quality of care in U.S. hospitals. *Health Aff (Millwood)* 2010; Sep;29(9):1593-9.

For peer review only - http://bmjopen.bmj.com/site/about/guidelines.xhtml

8. Surgical Safety Web Map. Available at: http://maps.cga.harvard.edu:8080/Hospital/. Accessed 07/12, 2012.

9. Kwok AC, Frank LM, Baltaga R, Lipsitz S, Merry AF, Dziekan G, et al. Implementation of the World Health Organization Surgical Safety Checklist, including introduction of pulse oximetry, in a resource-limited setting. *Annals of Surgery* 2013;257(4):633-9.

10. van Klei WA, Hoff RG, van Aarnhem EE, Simmermacher RKJ, Regli LPE, Kappen TH, et al. Effects of the introduction of the WHO “Surgical Safety Checklist” on in-hospital mortality: A cohort study. *Annals of Surgery* 2012;255(1):44-9.

11. Fourcade A, Blache J-, Grenier C, Bourgain J-, Minvielle E. Barriers to staff adoption of a surgical safety checklist. *BMJ Quality and Safety* 2012;21(3):191-7.

12. Levy SM, Senter CE, Hawkins RB, Zhao JY, Doody K, Kao LS, et al. Implementing a surgical checklist: More than checking a box. *Surgery (United States)* 2012;152(3):331-6.

13. Yuan CT, Walsh D, Walsh JL, Alpern R, Shakpeh J, Bradley EH. Incorporating the world health organization surgical safety checklist into practice at two hospitals in Liberia. *Joint Commission Journal on Quality and Patient Safety* 2012;38(6):254-60.

14. Bosk CL, Dixon-Woods M, Goeschel CA, Pronovost PJ. The art of medicine. Reality check for checklists. *The Lancet* 2009;374(9688):444-5.

15. Dixon-Woods M, Suokas A, Pitchforth E, Tarrant C. An ethnographic study of classifying and accounting for risk at the sharp end of medical wards. *Soc Sci Med* 2009; Aug;69(3):362-9.

16. McCarthy JC, Wright PC, Monk AF, Watts LA. Concerns at work: Designing useful procedures. *Human-Computer Interaction* 1998;13:433-57.

17. Vats A, Vincent CA, Nagpal K, Davies RW, Darzi A, Moorthy K. Practical challenges of introducing WHO surgical checklist: UK pilot experience. *BMJ* 2010;340.

For peer review only

For peer review only - http://bmjopen.bmj.com/site/about/guidelines.xhtml

18. Conley DM, Singer SJ, Edmondson L, Berry WR, Gawande AA. Effective surgical safety checklist implementation. *J Am Coll Surg* 2011;212(5):873-9.

19. Whyte S, Cartmill C, Gardezi F, Reznick R, Orser BA, Doran D, et al. Uptake of a team briefing in the operating theatre: A Burkean dramatistic analysis. *Social Science and Medicine* 2009;69(12):1757-66.

20. Safe Surgery in Africa. Available at: http://www.who.int/patientsafety/news/sssl_articles/en/index.html. Accessed 7/7, 2012.

21. Dixon-Woods M. What can ethnography do for quality and safety in health care?. *Qual Saf Health Care* 2003; Oct;12(5):326-7.

22. Attride-Stirling J. Thematic networks: An analytic tool for qualitative research. *Qualitative Research* 2001;1(3):385,405; 385.

23. Charmaz K. *Constructing grounded theory: A practical guide through qualitative analysis.* London: Sage; 2006.

24. WHO World Alliance for Patient Safety. Implementation manual WHO Surgical Safety Checklist (first edition). ; 2008. Report No.: WHO/IER/PSP/2008.05.

25. Hsia RY, Mbembati NA, MacFarlane S, Kruk ME. Access to emergency and surgical care in sub-Saharan Africa: The infrastructure gap. *Health Policy Plan* 2012;27(3):234-44.

26. Kotagal M, Lee P, Habiyakare C, Dusabe R, Kanama P, Epino HM, et al. Improving quality in resource poor settings: Observational study from rural Rwanda. *BMJ* 2009;339:1311-3.

27. Funk LM, Weiser TG, Berry WR, Lipsitz SR, Merry AF, Enright AC, et al. Global operating theatre distribution and pulse oximetry supply: An estimation from reported data. *The Lancet* 2010;376(9746):1055-61.

28. Deckelbaum DL, Ntakiyiruta G, Liberman AS, Razek T, Kyamanywa P. Augmenting surgical capacity in resource-limited settings. *The Lancet* 2011;.

For peer review only

For peer review only - http://bmjopen.bmj.com/site/about/guidelines.xhtml

29. Lavy C, Sauven K, Mkandawire N, Charian M, Gosselin R, Ndihokubwayo JB, et al. State of surgery in tropical africa: A review. *World J Surg* 2011;35:262-71.

30. De Vries EN, Prins HA, Crolla RMPH, Den Outer AJ, Van Andel G, Van Helden SH, et al. Effect of a comprehensive surgical safety system on patient outcomes. *N Engl J Med* 2010;363(20):1928-37.

31. Whyte S, Lingard L, Espin S, Baker GR, Bohnen BA, Orser B, et al. Paradoxical effects of interprofessional briefings on OR team performance. *Cognition, Technology and Work* 2008;10(4):287-94.

32. Dixon-Woods M, Leslie M, Tarrant C, Bion J. Explaining Matching Michigan: an ethnographic study of a patient safety program. *Implement Sci* 2013; Jun 20;8(1):70.

33. Wilson D. Who Guards the Guardians? Ian Kennedy, Bioethics and the 'Ideology of Accountability' in British Medicine. *Social History of Medicine* 2011; July 01;.

34. Aiken AM, Karuri DM, Wanyoro AK, Macleod J. Interventional studies for preventing surgical site infections in sub-Saharan Africa - A systematic review. *International Journal of Surgery* 2012;10(5):242-9.

35. Siddiqi K, Newell J, Robinson M. Getting evidence into practice: What works in developing countries?. *International Journal of Quality in Health Care* 2005;17:447-54.

36. Bradley EH, Yuan CT. Quality of care in low- and middle-income settings: What next?. *International Journal for Quality in Health Care* 2012;24(6):547-9.

37. Edmondson AC. Speaking up in the operating room: How team leaders promote learning in interdisciplinary action teams. *Journal of Management Studies* 2003;40(6):1419-52.

38. Dixon-Woods M, Bosk CL, Aveling EL, Goeschel CA, Pronovost PJ. Explaining Michigan: developing an ex post theory of a quality improvement program. *Milbank Q* 2011; Jun;89(2):167-205.

39. World Health Organization Global Patient Safety Challenge. Available at: http://www.who.int/patientsafety/challenge/en/. Accessed 06/30, 2013.

For peer review only - http://bmjopen.bmj.com/site/about/guidelines.xhtml

40. McCulloch P, Rathbone J, Catchpole K. Interventions to improve teamwork and communications among healthcare staff. *British Journal of Surgery* 2011;98(4):469-79.

41. Ider B-, Adams J, Morton A, Whitby M, Muugolog T, Lundeg G, et al. Using a checklist to identify barriers to compliance with evidence-based guidelines for central line management: A mixed methods study in Mongolia. *International Journal of Infectious Diseases* 2012;16(7):e551-7.

42. Walker IA, Merry AF, Wilson IH, McHugh GA, O'Sullivan E, Thoms GM, et al. Global oximetry: An international anaesthesia quality improvement project. *Anaesthesia* 2009;64(10):1051-60.

For peer review only

~~05/07/2013~~

~~Reality check for~~A qualitative study comparing experiences of the surgical safety checklist~~? A qualitative study comparing~~ in hospitals in high- and low-~~-~~income countries

Emma-Louise Aveling,[1*] Peter McCulloch,[2] Mary Dixon-Woods [3~~*~~]

[1] Research Associate, Department of Health Sciences, University of Leicester, 22-28 Princess Road West, Leicester LE1 6TP

[2] Clinical Reader in Surgery, Nuffield Department of Surgical Science, University of Oxford

[3]Professor of Medical Sociology, Department of Health Sciences, University of Leicester, ~~,~~22-28 Princess Road West, Leicester LE1 6TP

*Corresponding author: ~~Mary Dixon-Woods~~

Emma-Louise Aveling, Department of Health Sciences, University of Leicester, 22-28 Princess Road West, Leicester LE1 6TP, UK

Email: ~~md11~~eea5@le.ac.uk; Tel: +44116 ~~229 7262~~252 3287

For peer review only

For peer review only - http://bmjopen.bmj.com/site/about/guidelines.xhtml

05/07/2013

Article summary

ARTICLE FOCUS

- Bold claims have been made for the ability of the WHO surgical checklist to reduce surgical morbidity and mortality and improve patient safety.
- We aimed to identify and compare influences on checklist implementation and compliance in case study operating theatres in thetwo UK hospitals and Africaone African hospital.

KEY MESSAGES

- Consistent use, completion and fidelity of checklist deployment isare not straightforward in any sitesetting, but appearsmay be higher in the two UK hospitals compared with the hospital in a low-income settingcountry.
- Contrary to claims in early studies of the checklist, additional resources and changes to clinical systems may be needed to secure compliance with the checklist and its principles in low-income countriescontexts.
- ImprovementsThe checklist is no magic bullet; improvements in communication and quality of team interactions do not automatically follow theits introduction of a surgical checklist. Indeed,in any setting, and the checklist may indeed introduce new, unintended risks in low-income settings.

STRENGTHS AND LIMITATIONS

- Strengths: detailed, first-hand observations of the implementation of the surgical checklist in diverse settings; identification of key lessons of practical value in securing the benefits of the checklist and avoiding unintended consequences, especially in low-income countries.
- Limitations: data on outcomes were unavailable; observations conducted at different stages of implementation; some evidence of observer effect; limited number of sites; details of training were unreliable.

For peer review only - http://bmjopen.bmj.com/site/about/guidelines.xhtml

05/07/2013

ABSTRACT

Objective: Bold claims have been made for the ability of the WHO surgical checklist to reduce surgical morbidity and mortality and improve patient safety regardless of setting. Despite evidence of difficulties in checklist compliance in high income countries, little is known about how far the challenges faced by low income countries are the same or different. We aimed to identify and compare influences on checklist implementation and compliance in operating theatres in the UK and Africa.

Design: Ethnographic study involving observations, interviews, and collection of documents. Thematic analysis of data.

Setting: Operating theatres in one African university hospital and two UK university hospitalhospitals.

Participants: 112 hours of observations were undertaken. Interviews with 39 theatre and administrative staff were conducted.

Results: In Many staff saw value in the checklist in both the UK and African hospitals. Some resentment was present in all settings, compliance was adversely affected bylinked to conflicts between the philosophy behind the checklist and the realities of local cultural and, social context. Checklist complianceand economic contexts. Compliance – involving use, completeness and fidelity - was considerably higher, though not perfect, in a high-incomethe UK setting, where itsettings. In these hospitals, compliance was supported by established structures and systems for accountability and audit, and was not significantly undermined by major resource constraints. Resentment; the same was not true of the checklist as unnecessary was noted in all settings.low-income context. Hierarchical relationships were also a major barrier to implementation in all settings, but were more marked in the low-income African hospital.setting. Introducing a checklist in a professional environment characterised by a lack of accountability and transparency could make staff feel jeopardised legally, professionally, and personally, and it encouraged them to make misleading records of what had actually been done.

Conclusion: Surgical checklist useimplementation is likely to be optimised, regardless of setting, when used as one tool in multi-faceted cultural and organisational programmes to strengthen patient safety. It cannot be assumed that the introduction of a checklist will automatically lead to improved communication and clinical processes.

For peer review only - http://bmjopen.bmj.com/site/about/guidelines.xhtml

05/07/2013

INTRODUCTION

Surgery provides immediate, transformative treatment for many conditions, usually in a single episode, and will remain a vital part ofkey therapeutic strategy in all countries. Surgery is, unfortunately, also a major source of avoidable morbidity and mortality worldwide,[11, 2] though substantial improvements can be achieved by reducing variation in the reliability of surgical care processes..[3] Checklists are increasingly promoted as a way to deliver such reliability, following athe pilot study of the World Health Organization's (WHO) Surgical Safety Checklist (Box 1) which reported important reductions in mortality and complications in surgical patients.[24] The checklist involves 19 separate items to be checked at three distinct points: the Sign In, before induction of anaesthesia; the Time Out, before skin incision; and the Sign Out, at the end of the procedure.

One important and unusual feature of the WHO checklist is its claim to universality. The checklist pilot study[24] was distinctive for its being conducted in high, middle and lower income settings, including one African hospital. It concluded that the checklist programme could "improve the safety of surgical patients in diverse clinical and economic environments" (p496). It also suggested that implementation was neither costly or lengthy, proposing that only two items – pulse oximetry and prophylactic antibiotics - would involve commitment of significant resources; both were reported to be available at all sites in the study (including the low-income country hospitals) in the study,), though used inconsistently.

A second important feature of the WHO checklist is that it combines checks for technical items (such as administration of antibiotics and use of pulse oximeters) with other so--called "non-technical" items (such as team introductions and confirmations of procedures) whose principal purpose is to promote aspects of teamwork, communication and situational awareness. The surgical checklist's inclusionInclusion of the non-technical items in the checklist was influenced by prior research demonstrating an association between team practices (e.g. communication practicesbehaviours) and improved safety processes and attitudes.[3, 45, 6] Performing the checklist is also envisioned as a non-technical intervention in its own right: the checks are to be performed orally, and are, according to the checklist pilot study, "intentionally designed to create a collective awareness among surgical teams of whether safety processes are being completed" (p497).[24]

The potential for surgical safety checklists to improve safety and outcomes and generate substantial cost savings[57] has attracted global interest. In part owing to the WHO's Safe Surgery Saves Lives campaign, around 1800 institutions are now reported to be using the checklist worldwide.[68] However, Studies in high- and low-income settings have continued to report positive impacts on surgical outcomes, but the size of the reported effects on patient safety outcomes varies across and within settings, possibly because of variability in compliance.[7 99-13] Compliance can be heuristically distinguished into three dimensions: use, completeness and fidelity. Use refers to whether the checklist is used at all; completeness the extent to which it is completed in full, without items being skipped; and fidelity the extent to which items are performed as intended, with items ticked as

For peer review only - http://bmjopen.bmj.com/site/about/guidelines.xhtml

complete only when checks have genuinely been made, at the right time, and in communication with the whole team.

~~Implementing~~The checklist pilot study[4] and subsequent studies of resource-poor settings[9] were focused on assessment of outcomes, and did not characterise the implementation processes and characteristics of contexts likely to optimise checklist compliance. Yet implementing checklists~~ and~~, securing compliance and replicating positive outcomes necessarily ~~requires~~require an appreciation of ~~sociocultural~~socio-cultural context.[~~10~~14] Healthcare practitioners do not blindly follow procedures; rather, ~~implementation~~compliance often depends on workers' perceptions of effectiveness, relevance and rationale, their efforts to juggle competing professional, moral and social norms and demands, and the institutional and socio-economic contexts in which they work.[~~11, 12~~15, 16] ~~However, the focus of the checklist pilot study[2] on reporting outcomes made it difficult to identify the implementation processes and characteristics of contexts likely to optimise the impact of checklist introduction. Understanding what they are and whether they differ across contexts is important to attempts to replicate positive effects in diverse settings. Studies of the checklist~~ Studies of checklist implementation in high-income countries suggest that challenges to compliance are linked to organisational safety culture,[~~10~~14] and may include hierarchical structures, dismissive attitudes, absence of key team members, and hurrying through checks.[~~13-15~~17-19] Yet study of the checklist in low-income countries has been largely neglected. This is an important omission: complications and mortality following surgery in sub-Saharan Africa remain far higher than in developed countries[1] and almost 200 registered institutions in Africa are now reported to be using the checklist.[~~16~~20] The specific challenges likely to be faced in such settings remain ~~largely unknown.~~under-researched. We aimed to identify and compare influences on checklist implementation and compliance in ~~case study~~ operating theatres in ~~the UK~~hospitals in high-income and ~~Africa~~low-income countries.

METHODS

Our study design involved ethnographic case studies[~~17~~21] in three hospitals: two in the UK (anonymised as Amfield and Tolgrave) and one (anonymised as Mbile) in a low-income sub-Saharan African country. The UK centres were two large university hospitals in major urban settings that were participating in a larger research project on culture and behaviour relating to patient safety. The African centre was a university hospital serving a mixed urban and rural population of approximately 4-5 million. All three hospitals had adopted, as official policy, a checklist based on the WHO template. The checklist had been mandated for use in all UK hospitals by the National Patient Safety Agency two years before our study; in Mbile, it was introduced one year before as part of an externally funded international collaboration to improve ~~patient~~surgical safety. Given the sensitivity of some findings, we do not provide further details that could make participating sites or individual staff identifiable. Ethical approvals were obtained for the study in the UK and in the African site.

Data collected across all three sites comprised: i) ~~Semi~~semi-structured interviews with anaesthetists, surgeons, theatre staff, management and administrative staff~~ covering~~, and covered experiences of checklist implementation, institutional context, and management of patient safety. Interviews were conducted with informed consent from participants and were recorded~~ and~~, translated (where required) and transcribed; ii) non-standardised observations and informal discussions with staff in operating rooms, recorded as field-notes in notebooks and later elaborated more fully; iii) collation

For peer review only - http://bmjopen.bmj.com/site/about/guidelines.xhtml

~~05/07/2013~~

of relevant documentation. Observations were conducted in all four operating rooms in the African site. In the UK sites, observations were undertaken in a subset of theatres that covered a diversity of surgical specialities (including general, orthopaedic, oral-maxillofacial and vascular surgery). Interviewees were recruited after a period of observations had been completed, allowing the researchers to familiarise themselves with the setting and the organisation of theatres and theatre teams. Recruitment was guided by purposive sampling so that the sample was diverse in terms of seniority of staff (trainees to consultants and theatre managers), sub-specialty and disciplines (see Table 1). No one approached declined to be interviewed (although one staff member declined to have her interview recorded on religious grounds). ELA collected the data in two sites; a second observer (see acknowledgements) collected data in one of the UK sites. ELA analysed the data from all three sites. ELA and MDW independently reviewed a sample of data and together came to agreement on interpretation.

Data were analysed thematically,[~~18~~22] supported by Nvivo software, and guided but not constrained by sensitising concepts derived from the research questions.[~~19~~23] A single coding framework was iteratively developed, refined and applied to all three data sets. Data were initially coded into basic-themes within three global categories - implementation barriers, facilitators and contextual characteristics. In each category, basic codes were grouped into organising~~-~~ themes, and patterns in different sites were identified, compared and contrasted, paying attention to interrelations between themes. In addition, we analysed data from each site to identify patterns of use, completeness and fidelity of checklist compliance.

RESULTS

We interviewed 39 staff: 19 in Mbile and 20 across the two UK hospitals (see Table ~~2~~1). A total of 112 hours of observations were undertaken: 60 hours over two weeks in Mbile, 28 hours over four days in Amfield and 24 hours over three days in Tolgrave.

< TABLE 1 ABOUT HERE>

In all three sites, checklist implementation strategies included staff training, in situ demonstrations and awareness-raising at departmental meetings. In Mbile, training was assisted by two different international organisations, but surgeons were trained separately from other staff. This resulted in some mixed messages being circulated within the operating department.

Though in all ~~sites~~three hospitals some staff attributed initial problems with checklist compliance to lack of awareness or understanding, most staff in both the UK and African hospitals could describe broadly how it was *intended* to be used. In all ~~sites~~hospitals, many staff saw considerable value in the checklist. UK staff tended to emphasise prevention of rare but potentially catastrophic errors, such as wrong site surgery, and also suggested checklist use could ~~also~~ improve communication and teamwork with “no hierarchy” (Amfield, anaesthetist).

> *We’re trying to prevent what are usually rare errors, rare mistakes, you know, the majority of the things on that checklist are done most of the time without the checklist, but every now and then [...] you forget to check if you’re operating on the right leg and not the left leg, and*

For peer review only - http://bmjopen.bmj.com/site/about/guidelines.xhtml

For peer review only

05/07/2013

that's rare, but on very rare occasions it then leads to a disaster. [Amfield, Anaesthetist, U021]

It's not something fluffy and friendly, it is actually functional and it is about all respecting one another [Tolgrave, Anaesthetist, U014]

Mbile staff reported that few other protocols or standardised checks were in place in their hospital; some therefore welcomed the prompting and structure provided by the checklist. In contrast to the UK sites, they emphasised the checklist's value in catching more common mistakes – such as forgetting prophylactic antibiotics – but did not mention a role for the checklist in undermining hierarchies or improving teamwork.

Whenever we do the checklist we identify that some things have been missed [Mbile, Anaesthetist, A005]

Compliance

Nurses or theatre practitioners in all three hospitals had primary responsibility for initiating use of each checklist section, but differences in the way the checks were performed were evident. In UK hospitals, staff performed checks out loud in front of all team members who were present. In Mbile, checks were undertaken by an individual nurse, who ticked boxes based on his/her perceptions of what had happened or quietly checked on a one-to-one basis whether designated individuals had remembered specific tasks.

[During the procedure] I see the nurse filling in the checklist, which he then puts on top of the cupboard. He doesn't speak to anyone in the process, except to talk quietly with one of the surgical residents. [Mbile fieldnotes]

Though our study was not designed as a systematic audit, interviews and observations suggested that checklist use, completion, and fidelity, while not perfect, were more consistent in UK settings compared with Mbile. In the UK hospitals, the checklist was *used* in all procedures that we observed, documentation was fully *completed*, and interviewees reported internal audits showing checklist use in over 90% of procedures. *Fidelity* was more variable: in some instances teamwork was undermined by staff being distracted, dismissive, or absent during checks. During Time Out, staff often continued with other preparatory tasks, meaning it was not always the "moment of silence it's supposed to be" (nurse, Tolgrave). Sign In, supposed to be done before induction of anaesthesia, was often performed at the same time as the Time Out when the patient was already anaesthetised. Sign Out was often rushed or cursorily performed, though equipment counts were extremely thorough.

The scrub nurse and the student Operating Department Practitioner (ODP) are sort of removed, standing over by their metal trolley preparing some of the equipment and talking; they're not really listening to the rest of the group [surgeon, anaesthetist, ODP] who are crowded around patient (who's already anaesthetised), but they answer the questions when they're called to, and they do all the checks. [Amfield fieldnotes]

At the end of the operation, just as the patient is being moved from the operating table onto a trolley, and they're waiting for him to wake up [..] the anaesthetist says "oh, we haven't

For peer review only - http://bmjopen.bmj.com/site/about/guidelines.xhtml

done the sign out, oh, we should do the sign out". By this point the surgeon's already left the theatre [Amfield fieldnotes]

In Mbile, *use* of the checklist was highly inconsistent: participants' estimates ranged from 'always' to 'hardly ever', and direct observations induced a Hawthorne effect, where checklist use increased from few procedures early in our observation period to all procedures by the final two days. When staff were under pressure due to staff shortages, emergencies, or lengths of shifts (up to 36 hours) not seen in the UK, the checklist was apt to be abandoned altogether.

Sometimes it's difficult to use this [checklist], due to staff overload, so sometimes if there is an emergency case, they may not fill it in. Rather than fill it in, they might just get the instruments for those guys because of the urgency of the case [Mbile, Theatre ~~Nurse~~nurse, A011]

Completeness was both reported and observed to be highly variable in Mbile, with no improvement over the period of observations. *Fidelity* was also problematic in Mbile. Check-boxes were often ticked without the requisite information having been obtained or the tasks to which they referred undertaken, and the timing of checks was haphazard. Sometimes the nurse ticked the Sign-In and Time-Out checkboxes when the procedure was already underway, rendering them useless. Team introductions were never performed, yet the box was always ticked. Sometimes the Sign-Out boxes were ticked before the procedure was completed. This meant that equipment counts were ticked as complete when equipment was still in use, and specimens were recorded as correctly labelled before they had been removed from the patient. If equipment counts were performed at all in Mbile, they tended to be unreliable, completed in silence (not out loud as specified by the WHO guidance) by a single nurse at variable points during the procedure using a form that did not list all relevant equipment.

[During the procedure] I check the checklist: the nurse had (at some point before the end of the operation) filled the entire thing in, including "specimen is correctly labelled" – even though the specimen-to-be was still inside the patient. [Mbile fieldnotes]

The nurse fills in the back side of the form (the equipment count chart), even though all the equipment used in the operation has been cleared away, and completes the sign-out column of the checklist, without talking to anyone. [Mbile fieldnotes]

Factors influencing checklist implementation

Some reasons for variable compliance were similar between the UK and African sites; others were different, and some problems that appeared to be the same had different underlying mechanisms. A shared barrier to consistent compliance across the UK and ~~the~~ African settings was a perception among some that the checklist was futile, or that some checks (particularly the non-technical ones) were a waste of time.

The anaesthetist refuses to say his name. He says "I think we all know each other by now [...] it's ridiculous" [Amfield fieldnotes]

For peer review only - http://bmjopen.bmj.com/site/about/guidelines.xhtml

No, no, no [we don't do introductions] We know each other! I know the anaesthetist, I know the nurse. [Mbile, Surgeon, A017]

Perceptions ~~of pointlessness of~~that some elements of the checklist were pointlessness were partly based on the (demonstrably false) view among some staff at all three hospitals that incidents the checklist was designed to prevent would never happen in their environments. In all ~~sites~~hospitals the persuasive power of sentinel events – even very serious incidents (including, in Mbile, major operations on the wrong patients and a wrong site surgery in Amfield) – was variable.

I can clearly remember two occasions where the surgeon was just very dismissive and it was like, 'oh I'm not doing this, I don't need to do this, I'm not' you know... and literally just walked away. [Amfield, Operating Department Practitioner, U020]

Even though training on the checklist was given for surgeons, they don't use it, they don't believe in this bit of paper, because mostly they said, 'we don't mistake the identity of the patient, it doesn't happen that we get the wrong patient' [Mbile, Theatre nurse, A012]

Differences in factors influencing compliance, and their underlying mechanisms, derived from the very different contexts of implementation. Perhaps the most obvious difference between the settings concerned material resources. In all settings, the technical items were broadly accepted as established good practice. In the UK hospitals, equipment and medicines were available to address these items.

Mbile's infrastructure was very different. The electricity supply, for example, was unreliable; even basic equipment, such as gauze, was not reliably available. Such resource shortages meant that it was not always possible to comply with the technical items on the checklist, yet nurses often ticked the boxes nonetheless. For example, there were no surgical markers at Mbile hospital, but documentary analysis showed the question 'is the site marked?' was always ticked 'yes'. The box for prophylactic antibiotics was also among those most consistently completed ('yes' or 'not applicable' were usually ticked), but, with only a limited range of antibiotics available and no hospital policy regarding administration of prophylactic antibiotics, doctors sometimes delayed antibiotic use in case of subsequent post-operative infections. There were too few pulse oximeters available for the number of operations being performed. Anaesthetists were fully aware of the value of pulse oximeters, but were often unable to access one; absence of the device rarely led to cancellation of an operation.

We don't have too many types of antibiotics [...] if we have given [the patient] prophylactic antibiotics and organisms have already developed some resistance to the antibiotics, how are we going to treat this guy if he develops some type of infection later on? That is the problem [...] but we really don't have single view in this issue, I mean, some people give antibiotics, some people don't give, it's not a standardised policy [Mbile, Surgeon, A019]

In set-ups like ours, for example if there is no pulse oximetry, [..] if you consider it as a must we're going to lose many people because often we don't have this pulse oximetry. So if you say now I'm not going to anaesthetise a patient because I have no pulse oximeter definitely

For peer review only - http://bmjopen.bmj.com/site/about/guidelines.xhtml

we will lose many patients that could have been helped if you operate on them without pulse oximeter. [Mbile, Anaesthetist, A002]

The non-technical items on the checklist were far less dependent on material resources, yet they were much more likely to attract criticism and scepticism from staff. Some staff in all hospitals (UK and African) objected to the performance of the checklist in its entirety; more often, specific items were the subject of complaint. In the UK sites, a recurring objection was that the checklist duplicated pre-existing systems and checks, including, for example, long-standing equipment counting procedures. Perceptions of “duplicative” or “useless” checks contributed to the view of some in all settings that the checklist was an illegitimate bureaucratic intrusion, and resentment towards it intensified during busy periods, emergencies or staff shortages. The most deeply felt and repeatedly vocalised objections came from surgeons. Given strongly hierarchical team dynamics in both the UK and ~~Africa~~African hospitals, surgeons’ behaviour and attitudes in relation to the checklist were highly influential~~, making~~. This made it difficult for others to directly challenge defiant or dismissive surgeons.

I think it’s difficult because there is a hierarchy within the theatre complex, or within the medical profession~~,~~ full stop, and the theatre staff might feel intimidated by some of the environments they are in. Some of them are forceful enough and have a voice, but others are quite timid and probably quite reluctant to actually speak up. [Tolgrave, Anaesthetist, U012]

The surgeon may not volunteer to cooperate to fill that checklist. [...] The nurse may stop her work, or may do it without filling the checklist. It may depend on the confidence of the nurse. [Mbile, Theatre Nurse, A013]

Despite these objections, use of the checklist was high in the UK hospitals. Staff in these hospitals had been highly sensitised to issues of patient safety through exposure to their institutions’ policies and, though they did not see all patient safety interventions as positive, they felt that they had “a microscope on us about patient safety” (Tolgrave, nurse). Audit and feedback of data on checklist use and relevant outcomes (e.g. infection rates) were used to support checklist implementation and were tied to sanctions, which could include disciplinary action against staff who refused to comply.

You know I do not mind being part of any audit at all because I think it’s great, we won’t find anything or improve anything unless we do. [Tolgrave, ODP, U001]

~~I guess really just the fact that we have got a clearly focused campaign that says we will improve quality and safety, it’s making everyone step up to the plate and consider what they are doing and being audited on it. [Tolgrave, Anaesthetist,U014]~~

We have to be careful because you know, it’s in our registration as well to follow this protocol isn’t it, so [even] if the surgeon is not keen to do it, we have to tell them, you have to stop and do it [Tolgrave, ODP, U 008]

These UK audits were not, however, perfect instruments, not least because they generally failed to capture *how* checklists were being used. Beyond~~ these~~ formal arrangements, some local leaders acted as champions for the checklist. Surgeons, anaesthetists and senior nurses in both UK

For peer review only

For peer review only - http://bmjopen.bmj.com/site/about/guidelines.xhtml

settingshospitals took on this role, and were important in leading by example and supporting junior or non-surgical staff when there was "push-back".

We are there to iron out any problems, or if anybody gives them grief we can go in and fire it back if needed. [Tolgrave, Site coordinator, 002]

In Mbile, introducing the checklist involved much more far-reaching change than in the UK. The absence of an established tradition of equipment counts, for example, meant that the checklist was a much more disruptive and demanding intervention in the African setting. Items that were non-controversial in a UK setting, such as documenting patient consent, had different meaning in a context where patients often lacked literacy and surgery was considered a privilege. The surgical checklist was one of the first explicit patient safety interventions introduced in Mbile. Staff had little prior exposure to the ideas and principles of the science of safety, and systems to support patient safety were poorly developed. The hospital lacked an established culture of audit; there was little routine collection of clinical data and staff had no clear sense of the impact of the checklist on patient safety. There was no monitoring of checklist use, and no consequences for non-compliance.

I asked every department [if they want to do an audit] and they have not volunteered […] I think they're busy. It is not familiar, clinical audit. Most health workers don't know what clinical audit means. [Mbile, Administrator, A001]

We really don't have the real number [for infection rates], how many patients have been affected, because we haven't applied those measurements, we really don't know [Mbile, Surgeon, A020]

It's ok, nothing happens. For those who don't fill in the checklist there is no problem, no consequence. [Mbile, Theatre nurse, A011]

Team dynamics were especially challenging in Mbile. Though local championing of the checklist was not absent, it was not highly visible either. Surgeons were the only doctors in the room: anaesthetists were not physicians, but held a BSc in anaesthesia. In observations, challenge over checklist use was very rarely witnessed. Wider societal hierarchies and cultural norms, including those relating to age, gender and education, resulted in particularly steep authority gradients. Nurses were often female and younger than the (exclusively) male surgeons, and had been socialised to be deferential and submissive. The working environment was described as lacking in transparency and accountability, particularly in relation to staff dismissals or promotions.

There were complaints that there is impartial [unfair] treatment between people. This is one of the problems from the managerial aspect. [Mbile, Anaesthetist,A 002 A002]

The external socio-legal context in Mbile was one that staff felt was at best unreliable. At worst it was seen corrupt and unjust, offering little protection to vulnerable, low-status individuals. Interviews suggested that challenging those in authority could therefore feel very risky and have unpredictable results, perhaps damaging career prospects or even worse (Box 2). In observations, challenge over checklist use was very rarely witnessed. The checklist thus introduced new, unintended risks for staff.

For peer review only - http://bmjopen.bmj.com/site/about/guidelines.xhtml

~~05/07/2013~~

<Box 2 about here>

DISCUSSION

The WHO's implementation manual identifies two purposes for the surgical checklist: ensuring consistency in patient safety and introducing (or maintaining) a culture that values achieving it.[~~20~~24] Our study suggests that hospitals in both high and low-income countries may experience challenges in delivering on these aims~~.~~, even when many staff (non-physicians in particular) can see value in the checklist. An important finding is the extent to which hospitals in both low and high-income countries encountered the same obstacles in implementing the checklist; the key differences related to contexts. Material contexts were perhaps most vividly distinct: resource shortages meant that it was often impossible to comply with the checklist's technical requirements in Mbile. Resource and infrastructure constraints in low-income countries have been well~~–~~-described previously,[~~21-23~~25-29] suggesting that the problems in our African study hospital were unlikely to be unique. Kwok and colleagues,[9] for example, similarly identified insufficient numbers of pulse oximeters as a barrier to checklist compliance, and suggest that the hospitals in the original pilot study were perhaps not representative of those in low-income settings that lack basic resources. Greater acknowledgement may therefore be needed that additional material resources may well be required to implement the checklist in low-come countries. Many of the non-technical items on the checklist ~~did~~do not, of course, require additional material resources, but rather changes in communication practices and teamwork. Hierarchical team dynamics undermining of safety were evident in all three hospitals in our study, but were more pronounced in the African setting. ~~Our findings raise questions about the claim in the pilot study of the checklist[2] that few additional resources or changes to clinical systems would be needed to implement the checklist. Our findings also challenge the~~The assumption that improvements in communication and team interactions automatically follow the introduction of a checklist~~.~~[~~2, 24~~4, 30] ~~Contrary to the pilot study hypothesis, introduction of the checklist did not "signify" "associated cultural changes"[2] (p.493); rather, our~~ is thus open to question. Our study points to a much more complex interrelation between checklist procedures, context, 'culture' and behavioural changes, and confirms that checklists should not be regarded as magic bullets.

Contextual differences meant that Mbile had much more to do to implement and secure compliance with the checklist, and it lacked features that supported implementation in the UK sites. Local policies, institutional focus and support, well-established audit and data collection systems, clear lines of accountability with consequences for staff and hospitals for non-compliance were all more pronounced (if not always perfect) in the UK hospitals than their African counterpart. They were important indirectly, by signalling the institutional priority given to patient safety,[~~14~~18] and directly (e.g. disciplinary action). Despite these advantages, the UK sites also~~ suffered from significant barriers to implementation, with~~, showed evidence of persisting problems of fidelity, audits that focused too much on documentation of box-ticking, and resentment of perceived top-down initiatives.

Our study produced some disquieting evidence that poor checklist implementation in low-income settings might not only fail to reduce patient safety risks, but also *introduce* new risks for staff and/or patients. Previous work has also shown that even in well-resourced settings, checklists may not consistently deliver positive effects, and may potentially produce some paradoxical or harmful

For peer review only - http://bmjopen.bmj.com/site/about/guidelines.xhtml

05/07/2013

effects including inhibition of team processes.[3, 255, 31] These may be even more consequential in a low -income setting. The style of checks in Mbile limited improvements in team communication, and was directly contrary to the design intent of creating collective awareness. The authoritarian status of the surgeon elite contributed to these unhelpful features of team dynamics, which were exacerbated by wider societal hierarchies and a lack of transparency in institutional processes. Though unhelpful team dynamics were also sometimes evident in the UK hospitals, introducing a checklist in a professional environment characterised by a lack of accountability and transparency raised additional ethical concerns in Mbile: –it made some staff feel jeopardised legally, professionally, and personally, and it encouraged them to make misleading records of what had actually been done. In this situationThus, although many staff welcomed attempts to introduce structure and standardisation to procedure the preparation and organisation of procedures, the benefits for patient safety were unclear, especially as no audit data were available to establish outcomes.

None of this is to deny the potential of the checklist to deliver significant benefits for the safety of patients in low-income settings, but it is to emphasise the need for a high level of sensitivity to cultural and economic contexts in the design and implementation of interventions. Introduction of the checklist may not, of itself, address underlying deficits in the clinical processes the checks cover. Workers cannot comply with checklist items that require material resources (e.g. antibiotics and pulse oximeters) that are not available in their organisations. Many non-technical items, of course, represent potentially low-cost ways to improve care and may thus be attractive for resource-constrained environments, particularly when many healthcare interventions are so expensive. But attempts to change the status quo in these settings should be informed by a sound evidence base, attention to unintended consequences and recognition of the influence of local histories.[32] Though the volume of research on patient safety in low-income countries is now increasing,[26, 2733, 34] little is known about which kinds of strategies are most effective in addressing patient safety issues in these settings.[2835]

Our study suggests that explicit interventional focus on improving team dynamics and communication may be even more critical in African healthcare settings than high-income countries. Professional groups – in whatever setting - should be trained together on the checklist, not separately as occurred in Mbile. Additional interventional focus on relevant clinical systems may also be necessary. For example, our findings suggest that where equipment count practices are not well established, focused training, agreed procedures and on-going support to implement these practices are needed if the use of checklists is to be meaningful. Our findings also reinforce lessons from improvement science more broadly concerning the importance of collection and feedback of data; in low-income settings where audit is not such an established feature of institutional governance, additional capacity building may be needed. [36]

The critical role of local leads in motivating the adoption of new ideas[2937] means that particular efforts must be made to secure buy-in from surgeons and to ensure that junior or non-medical staff are assured of institutional support. Multiple, senior champions from across the relevant disciplines is needed to ensure leadership is visible and persuasive. Another effective tactic may be to empower theatre staff to call on supportive senior surgeons ('champions') for back-up when they face 'push back' from other staff.[38] While high-level institutional strategies were seen by some as misdirected

For peer review only

For peer review only - http://bmjopen.bmj.com/site/about/guidelines.xhtml

or problematic in UK settings, the comparison with Mbile nonetheless underscores the value of all front-line staff having ~~high level institutional support and clear leadership for their efforts to improve safety~~support and clear leadership for their efforts to improve safety. Such institutional support should also include the judicious use of 'hard edges'[38] and mechanisms through which there are predictable consequences for staff – at all levels – when patient safety is jeopardised. As patient safety becomes more firmly established on local and national health agendas in low-income countries, for example through initiatives such as the WHO's Global Patient Safety Challenge,[39] institutional and infrastructural support for safety is likely to improve as well. Attention to strategies likely to improve implementation (Box 3) may help with this.

Our study has a number of limitations. It did not include data on outcomes. Mbile had only been ~~implementing~~using the checklist for one year, in contrast to two years in UK hospitals, so was at a different stage of implementation. Despite the importance of training to secure checklist compliance,[~~30~~40] the precise nature of what was provided in the three sites (especially in Mbile) was unclear, due to unreliable (and in some cases conflicting) accounts of events. A clear observer effect was found in Mbile, and may also have occurred in UK hospitals. Observations over a longer period in all sites might have identified more influences on checklist compliance. Nonetheless, our study has yielded important lessons for implementing surgical checklists ~~(Box 3).~~in different settings.

Our work demonstrates that assumptions about context are encoded into patient safety tools. Optimising checklist ~~use~~deployment requires careful analysis of these assumptions, and of the extent to which ~~these~~they match local set-ups or require the introduction of new systems and practices. It also demonstrates that changes in teamwork practices ~~will~~do not automatically follow checklist introduction, even where non-technical items are included, but rather require explicit, interventional focus. Checklists are neither a 'quick-fix' nor a tool that can be effectively implemented in isolation; in resource-constrained settings, they are especially unlikely to be free of costs and risks. Safety checklists are most likely to be effective and sustainable when implemented as part of broader, multi-faceted programs addressing social, behavioural, logistical and organisational issues,[~~10, 31-33~~14, 26, 41, 42] where there is strong institutional focus on patient safety, multi-disciplinary leadership, monitoring systems in place, and consequences at all levels for non-compliance.

<Box 3 about here>

For peer review only - http://bmjopen.bmj.com/site/about/guidelines.xhtml

For peer review only

~~05/07/2013~~

Role/ Profession	Mbile (African hospital)	Amfield and Tolgrave (UK hospitals)
Anaesthetists	4	4
Surgeons	6	3
Managers/administrators	4	4
Nurses and Theatre ~~staff~~Practitioners	5	9
Total	***19***	***20***

Table 1. Number of individual interviews by profession in African and UK sites

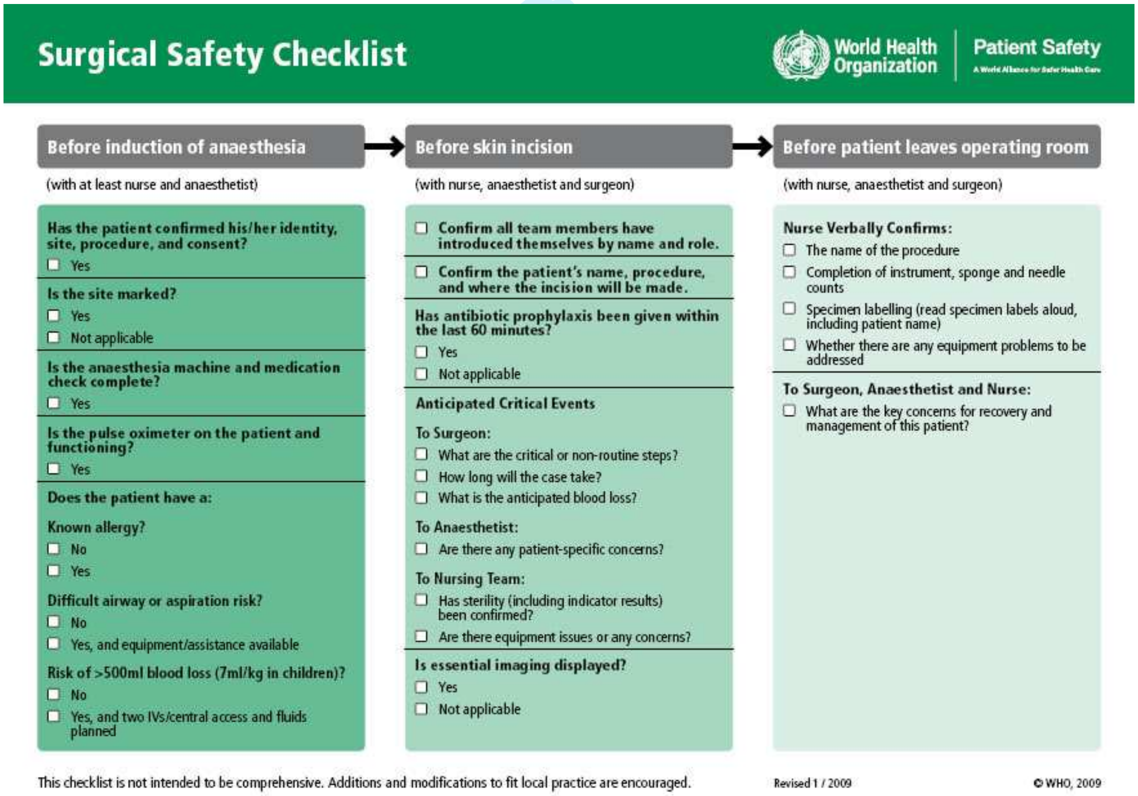

Surgical Safety Checklist

World Health Organization | Patient Safety

Before induction of anaesthesia

(with at least nurse and anaesthetist)

Has the patient confirmed his/her identity, site, procedure, and consent?
- ☐ Yes

Is the site marked?
- ☐ Yes
- ☐ Not applicable

Is the anaesthesia machine and medication check complete?
- ☐ Yes

Is the pulse oximeter on the patient and functioning?
- ☐ Yes

Does the patient have a:

Known allergy?
- ☐ No
- ☐ Yes

Difficult airway or aspiration risk?
- ☐ No
- ☐ Yes, and equipment/assistance available

Risk of >500ml blood loss (7ml/kg in children)?
- ☐ No
- ☐ Yes, and two IVs/central access and fluids planned

Before skin incision

(with nurse, anaesthetist and surgeon)

- ☐ **Confirm all team members have introduced themselves by name and role.**
- ☐ **Confirm the patient's name, procedure, and where the incision will be made.**

Has antibiotic prophylaxis been given within the last 60 minutes?
- ☐ Yes
- ☐ Not applicable

Anticipated Critical Events

To Surgeon:
- ☐ What are the critical or non-routine steps?
- ☐ How long will the case take?
- ☐ What is the anticipated blood loss?

To Anaesthetist:
- ☐ Are there any patient-specific concerns?

To Nursing Team:
- ☐ Has sterility (including indicator results) been confirmed?
- ☐ Are there equipment issues or any concerns?

Is essential imaging displayed?
- ☐ Yes
- ☐ Not applicable

Before patient leaves operating room

(with nurse, anaesthetist and surgeon)

Nurse Verbally Confirms:
- ☐ The name of the procedure
- ☐ Completion of instrument, sponge and needle counts
- ☐ Specimen labelling (read specimen labels aloud, including patient name)
- ☐ Whether there are any equipment problems to be addressed

To Surgeon, Anaesthetist and Nurse:
- ☐ What are the key concerns for recovery and management of this patient?

This checklist is not intended to be comprehensive. Additions and modifications to fit local practice are encouraged.

Revised 1 / 2009 © WHO, 2009

For peer review only - http://bmjopen.bmj.com/site/about/guidelines.xhtml

~~05/07/2013~~

Box 1: WHO Surgical Safety Checklist (~~permission to use Figure currently awaited~~reproduced with the permission of the WHO; available from http://www.who.int/patientsafety/safesurgery/tools_resources/SSSL_Checklist_finalJun08.pdf)

A patient admitted for cholecystectomy suffered hypoxic brain injury and died following surgery. Subsequently, two staff members (not the surgeon) were threatened with guns by the patient's family, who said the surgical team had killed the patient. The two staff members were later arrested and criminal charges brought against one of them. One of the questions asked during the police investigation was whether a pulse oximeter had been used. It had not: according to staff, no pulse oximeter was available for use, even though the checklist requiring use of this equipment was ~~official~~, officially, in use at the hospital ~~policy~~. Adoption of the checklist by the hospital, without adequate provision of the resources necessary for compliance, had left low-status front-line staff more socially and legally vulnerable, as it could be argued that they had failed to comply with hospital policy. Moreover, at all levels of the institution there was a great deal of uncertainty about staff rights and their employers' responsibilities in such circumstances. The accused staff were not provided with any legal representation for some weeks.

Box. 2: Description of events following an adverse event in Mbile, illustrative of the wider socio-legal context.

For peer review only - http://bmjopen.bmj.com/site/about/guidelines.xhtml

05/07/2013

- Checklist implementation will be optimised when part of a broader, multi-faceted cultural and organisational programme to strengthen patient safety
- Introduction of the checklist does not automatically lead to changes in teamwork and communication, despite the inclusion of non-technical items
  - Improvements in communication and teamwork require direct interventional focus
  - Whole team training (rather than separate training for different professional groups) is important
  - Hierarchical dynamics within the operating theatre may be particularly prominent in some low-income contexts, exacerbated by the wider socio-legal context
- Assumptions about implementation contexts (e.g. resources, policies, systems in place) encoded within the checklist require careful scrutiny
  - Performance of the checklist may not be the only change(s) required
  - In resource-constrained settings especially, existing systems and resources may need to be strengthened for the checklist to be used with completeness and fidelity
- Local leadership and championing of the checklist is important and needs to:
  - Include support from the highest institutional level
  - Include senior staff, particularly senior surgeons
  - Include leaders from all professional groups
  - Be visible in theatres
- Monitoring (including process and impact measures) must be in place and fed back to staff
- Poor compliance (use, completeness and/or fidelity) may not only fail to strengthen surgical safety – it may also introduce risks for staff and/or patients in some environments

For peer review only - http://bmjopen.bmj.com/site/about/guidelines.xhtml

05/07/2013

- Checklist implementation will be optimised when part of a broader, multi-faceted cultural and organisational programme to strengthen patient safety
- Introduction of the checklist does not automatically lead to changes in teamwork and communication, despite the inclusion of non-technical items. Hierarchical dynamics within the operating theatre may be particularly prominent in some low-income contexts, exacerbated by the wider socio-legal context
    - Improvements in communication and teamwork require direct interventional focus
    - Whole team training (rather than separate training for different professional groups) is important
- Assumptions about implementation contexts (e.g. resources, policies, systems in place) encoded within the checklist require careful scrutiny
    - Performance of the checklist may not be the only change(s) required
    - In resource-constrained settings especially, existing systems and resources may need to be strengthened for the checklist to be used with completeness and fidelity (e.g. provision of pulse oximetry)
- Local leadership and championing of the checklist is important and needs to:
    - Include support from the highest institutional level
    - Include senior staff, particularly senior surgeons
    - Include leaders from all professional groups
    - Be visible in theatres
- Monitoring (including process and impact measures) must be in place and fed back to all staff
- Poor compliance (use, completeness and/or fidelity) may not only fail to strengthen surgical safety - it may also introduce risks for staff and/or patients in some environments

Box 3. Lessons for checklist implementation in high- and low-income country settings

For peer review only - http://bmjopen.bmj.com/site/about/guidelines.xhtml

~~05/07/2013~~

Declarations

Acknowledgements: Our sincere thanks to the staff of all three hospitals for their time and participation in the study. Our thanks to Joel Minion, who collected the data from one of the UK sites. We would also like to thank Dr Wynne Aveling for the clinical expertise he offered during data collection.

Ethics: Ethical approval was granted by the Leicestershire, Northamptonshire and Rutland Research Ethics Committee (number 10/H0406/77), and the Institutional Ethical Review Board for research in the African site. All participants gave informed consent.

Funding: We thank the Higher Education Innovation Fund Impact Award; Wellcome Trust Senior Investigator Award WT097899MA; and the Department of Health Policy Research Programme (reference number 0770017) for providing funding for this study.

Role of sponsor: The funders did not have a role in study design; in the collection, analysis, and interpretation of data; in the writing of the report or the decision to submit the article for publication. The research was conducted independently of the funders.

Contributorship: ELA and MDW jointly conceived and designed the study with support from PM. ELA negotiated access, recruited participants and conducted the fieldwork in one UK site and the African site. ELA analysed the data from all three sites. ELA and MDW conducted the literature review, with additional suggestions from PM. ELA and MDW led the writing of the manuscript. PM contributed clinical expertise. All authors contributed to interpretation of the findings, contributed to drafting the manuscript and approved the final version. ELA is the guarantor.

Data access: ELA had full access to all of the data in the study and can take responsibility for the integrity of the data and the accuracy of the data analysis. MDW had full access to the data from the UK sites and the opportunity to access the African data. For ethical reasons full data were not shared with PM.

Data sharing: No additional data available.

Competing interest declaration: All authors have completed the Unified Competing Interest form at www.icmje.org/coi_disclosure.pdf (available on request from the corresponding author) and declare that (1) ELA, MDW, PM] have no relationships with [any companies] that might have an interest in the submitted work in the previous 3 years; (2) their spouses, partners, or children have no financial relationships that (3) ELA, MDW, PM have no non-financial interests that may be relevant to the submitted work.

For peer review only - http://bmjopen.bmj.com/site/about/guidelines.xhtml

~~05/07/2013~~

The Corresponding Author has the right to grant on behalf of all authors and does grant on behalf of all authors, a worldwide licence (http://www.bmj.com/sites/default/files/BMJ%20Author%20Licence%20March%202013.doc) to the Publishers and its licensees in perpetuity, in all forms, formats and media (whether known now or created in the future), to i) publish, reproduce, distribute, display and store the Contribution, ii) translate the Contribution into other languages, create adaptations, reprints, include within collections and create summaries, extracts and/or, abstracts of the Contribution and convert or allow conversion into any format including without limitation audio, iii) create any other derivative work(s) based in whole or part on the on the Contribution, iv) to exploit all subsidiary rights to exploit all subsidiary rights that currently exist or as may exist in the future in the Contribution, v) the inclusion of electronic links from the Contribution to third party material where-ever it may be located; and, vi) licence any third party to do any or all of the above. All research articles will be made available on an Open Access basis (with authors being asked to pay an open access fee—see http://www.bmj.com/about-bmj/resources-authors/forms-policies-and-checklists/copyright-open-access-and-permission-reuse). The terms of such Open Access shall be governed by a Creative Commons licence—details as to which Creative Commons licence will apply to the research article are set out in our worldwide licence referred to above.

For peer review only

For peer review only - http://bmjopen.bmj.com/site/about/guidelines.xhtml

~~05/07/2013~~

References

1. Bainbridge D, Martin J, Arango M, Cheng D, Evidence-based Peri-operative Clinical Outcomes Research (EPiCOR) Group. Perioperative and anaesthetic-related mortality in developed and developing countries: a systematic review and meta-analysis. *Lancet* 2012; Sep 22;380(9847):1075-81.

2. Anderson O, Davis R, Hanna GB, Vincent CA. Surgical adverse events: A systematic review. *Am J Surg* 2013; Apr 30;Epub ahead of print.

3. Kreckler S, Morgan RD, Catchpole K, New S, Handa A, Collins G, et al. Effective prevention of thromboembolic complications in emergency surgery patients using a quality improvement approach. *Bmj Quality & Safety* 2013; May 24;Epub ahead of print.

4. Haynes AB, Weiser TG, Berry WR, Lipsitz SR, Breizat AHS, Dellinger EP, et al. A surgical safety checklist to reduce morbidity and mortality in a global population. *N Engl J Med* 2009;260:491-9.

~~3. Pronovost P, Needham D, Berenholtz S, Sinopoli D, Chu H, Cosgrove S, et al. An intervention to decrease catheter-related bloodstream infections in the ICU. *N Engl J Med* 2006;355(26):2725-32.~~

~~4~~5. Lingard L, Regehr G, Orser B, Reznick R, Baker GR, Doran D, et al. Evaluation of a preoperative checklist and team briefing among surgeons, nurses, and anesthesiologists to reduce failures in communication. *Archives of Surgery* 2008;143(1):12-7.

~~5~~6. Leonard M, Graham S, Bonacum D. The human factor: The critical importance of effective teamwork and communication in providing safe care. *Quality and Safety in Health Care* 2004;13(SUPPL. 1):i85-90.

For peer review only

For peer review only - http://bmjopen.bmj.com/site/about/guidelines.xhtml

05/07/2013

6.7 Dixon-Woods M, Suokas A, Pitchforth E, Tarrant C. An ethnographic study of classifying and accounting for risk at the sharp end of medical wards. *Soc Sci Med* 2009; Aug;69(3):362-9.

7. McCarthy JC, Wright PC, Monk AF, Watts LA. Concerns at work: Designing useful procedures. *Human-Computer Interaction* 1998;13:433-57.

8. Semel ME, Resch S, Haynes AB, Funk LM, Bader A, Berry WR, et al. Adopting a surgical safety checklist could save money and improve the quality of care in U.S. hospitals. *Health Aff (Millwood)* 2010; Sep;29(9):1593-9.

98. Surgical Safety Web Map. Available at: http://maps.cga.harvard.edu:8080/Hospital/. Accessed 07/12, 2012.

9. Kwok AC, Frank LM, Baltaga R, Lipsitz S, Merry AF, Dziekan G, et al. Implementation of the World Health Organization Surgical Safety Checklist, including introduction of pulse oximetry, in a resource-limited setting. *Annals of Surgery* 2013;257(4):633-9.

10. van Klei WA, Hoff RG, van Aarnhem EE, Simmermacher RKJ, Regli LPE, Kappen TH, et al. Effects of the introduction of the WHO "Surgical Safety Checklist" on in-hospital mortality: A cohort study. *Annals of Surgery* 2012;255(1):44-9.

11. Fourcade A, Blache J-, Grenier C, Bourgain J-, Minvielle E. Barriers to staff adoption of a surgical safety checklist. *BMJ Quality and Safety* 2012;21(3):191-7.

12. Levy SM, Senter CE, Hawkins RB, Zhao JY, Doody K, Kao LS, et al. Implementing a surgical checklist: More than checking a box. *Surgery (United States)* 2012;152(3):331-6.

For peer review only

13. Yuan CT, Walsh D, Walsh JL, Alpern R, Shakpeh J, Bradley EH. Incorporating the world health organization surgical safety checklist into practice at two hospitals in Liberia. *Joint Commission Journal on Quality and Patient Safety* 2012;38(6):254-60.

14. Bosk CL, Dixon-Woods M, Goeschel CA, Pronovost PJ. The art of medicine. Reality check for checklists. *The Lancet* 2009;374(9688):444-5.

15. Dixon-Woods M, Suokas A, Pitchforth E, Tarrant C. An ethnographic study of classifying and accounting for risk at the sharp end of medical wards. *Soc Sci Med* 2009; Aug;69(3):362-9.

16. McCarthy JC, Wright PC, Monk AF, Watts LA. Concerns at work: Designing useful procedures. *Human-Computer Interaction* 1998;13:433-57.

~~14~~17. Vats A, Vincent CA, Nagpal K, Davies RW, Darzi A, Moorthy K. Practical challenges of introducing WHO surgical checklist: UK pilot experience. *BMJ* 2010;340.

~~15~~18. Conley DM, Singer SJ, Edmondson L, Berry WR, Gawande AA. Effective surgical safety checklist implementation. *J Am Coll Surg* 2011;212(5):873-9.

~~16~~19. Whyte S, Cartmill C, Gardezi F, Reznick R, Orser BA, Doran D, et al. Uptake of a team briefing in the operating theatre: A Burkean dramatistic analysis. *Social Science and Medicine* 2009;69(12):1757-66.

~~17~~20. Safe Surgery in Africa. Available at: http://www.who.int/patientsafety/news/sssl_articles/en/index.html. Accessed 7/7, 2012.

~~18~~21. Dixon-Woods M. What can ethnography do for quality and safety in health care?. *Qual Saf Health Care* 2003; Oct;12(5):326-7.

For peer review only

For peer review only - http://bmjopen.bmj.com/site/about/guidelines.xhtml

~~05/07/2013~~

~~19~~22. Attride-Stirling J. Thematic networks: An analytic tool for qualitative research. *Qualitative Research* 2001;1(3):385,405; 385.

~~20~~23. Charmaz K. *Constructing grounded theory: A practical guide through qualitative analysis.* London: Sage; 2006.

~~21~~24. WHO World Alliance for Patient Safety. Implementation manual WHO Surgical Safety Checklist (first edition). ; 2008. Report No.: WHO/IER/PSP/2008.05.

25. Hsia RY, Mbembati NA, MacFarlane S, Kruk ME. Access to emergency and surgical care in sub-Saharan Africa: The infrastructure gap. *Health Policy Plan* 2012;27(3):234-44.

26. Kotagal M, Lee P, Habiyakare C, Dusabe R, Kanama P, Epino HM, et al. Improving quality in resource poor settings: Observational study from rural Rwanda. *BMJ* 2009;339:1311-3.

~~22. Deckelbaum DL, Ntakiyiruta G, Liberman AS, Razek T, Kyamanywa P. Augmenting surgical capacity in resource-limited settings. *The Lancet* 2011;.~~

~~23~~27. Funk LM, Weiser TG, Berry WR, Lipsitz SR, Merry AF, Enright AC, et al. Global operating theatre distribution and pulse oximetry supply: An estimation from reported data. *The Lancet* 2010;376(9746):1055-61.

28. Deckelbaum DL, Ntakiyiruta G, Liberman AS, Razek T, Kyamanywa P. Augmenting surgical capacity in resource-limited settings. *The Lancet* 2011;.

~~24~~29. Lavy C, Sauven K, Mkandawire N, Charian M, Gosselin R, Ndihokubwayo JB, et al. State of surgery in tropical africa: A review. *World J Surg* 2011;35:262-71.

30. De Vries EN, Prins HA, Crolla RMPH, Den Outer AJ, Van Andel G, Van Helden SH, et al. Effect of a comprehensive surgical safety system on patient outcomes. *N Engl J Med* 2010;363(20):1928-37.

For peer review only - http://bmjopen.bmj.com/site/about/guidelines.xhtml

05/07/2013

2531. Whyte S, Lingard L, Espin S, Baker GR, Bohnen BA, Orser B, et al. Paradoxical effects of interprofessional briefings on OR team performance. *Cognition, Technology and Work* 2008;10(4):287-94.

2632. Dixon-Woods M, Leslie M, Tarrant C, Bion J. Explaining Matching Michigan: an ethnographic study of a patient safety program. *Implement Sci* 2013; Jun 20;8(1):70.

33. Wilson D. Who Guards the Guardians? Ian Kennedy, Bioethics and the 'Ideology of Accountability' in British Medicine. *Social History of Medicine* 2011; July 01;.

2734. Aiken AM, Karuri DM, Wanyoro AK, Macleod J. Interventional studies for preventing surgical site infections in sub-Saharan Africa - A systematic review. *International Journal of Surgery* 2012;10(5):242-9.

2835. Siddiqi K, Newell J, Robinson M. Getting evidence into practice: What works in developing countries?. *International Journal of Quality in Health Care* 2005;17:447-54.

2936. Bradley EH, Yuan CT. Quality of care in low- and middle-income settings: What next?. *International Journal for Quality in Health Care* 2012;24(6):547-9.

37. Edmondson AC. Speaking up in the operating room: How team leaders promote learning in interdisciplinary action teams. *Journal of Management Studies* 2003;40(6):1419-52.

3038. Dixon-Woods M, Bosk CL, Aveling EL, Goeschel CA, Pronovost PJ. Explaining Michigan: developing an ex post theory of a quality improvement program. *Milbank Q* 2011; Jun;89(2):167-205.

39. World Health Organization Global Patient Safety Challenge. Available at: http://www.who.int/patientsafety/challenge/en/. Accessed 06/30, 2013.

For peer review only

For peer review only - http://bmjopen.bmj.com/site/about/guidelines.xhtml

05/07/2013

40. McCulloch P, Rathbone J, Catchpole K. Interventions to improve teamwork and communications among healthcare staff. *British Journal of Surgery* 2011;98(4):469-79.

3141. Ider B-, Adams J, Morton A, Whitby M, Muugolog T, Lundeg G, et al. Using a checklist to identify barriers to compliance with evidence-based guidelines for central line management: A mixed methods study in Mongolia. *International Journal of Infectious Diseases* 2012;16(7):e551-7.

32. Kotagal M, Lee P, Habiyakare C, Dusabe R, Kanama P, Epino HM, et al. Improving quality in resource poor settings: Observational study from rural Rwanda. *BMJ* 2009;339:1311-3.

3342. Walker IA, Merry AF, Wilson IH, McHugh GA, O'Sullivan E, Thoms GM, et al. Global oximetry: An international anaesthesia quality improvement project. *Anaesthesia* 2009;64(10):1051-60.

1. Bainbridge D, Martin J, Arango M, Cheng D, Evidence-based Peri-operative Clinical Outcomes Research (EPiCOR) Group. Perioperative and anaesthetic-related mortality in developed and developing countries: a systematic review and meta-analysis. *Lancet* 2012; Sep 22;380(9847):1075-81.

2. Haynes AB, Weiser TG, Berry WR, Lipsitz SR, Breizat AHS, Dellinger EP, et al. A surgical safety checklist to reduce morbidity and mortality in a global population. *N Engl J Med* 2009;260:491-9.

3. Lingard L, Regehr G, Orser B, Reznick R, Baker GR, Doran D, et al. Evaluation of a preoperative checklist and team briefing among surgeons, nurses, and anesthesiologists to reduce failures in communication. *Archives of Surgery* 2008;143(1):12-7.

4. Leonard M, Graham S, Bonacum D. The human factor: The critical importance of effective teamwork and communication in providing safe care. *Quality and Safety in Health Care* 2004;13(SUPPL. 1):i85-90.

5. Semel ME, Resch S, Haynes AB, Funk LM, Bader A, Berry WR, et al. Adopting a surgical safety checklist could save money and improve the quality of care in U.S. hospitals. *Health Aff (Millwood)* 2010; Sep;29(9):1593-9.

6. Surgical Safety Web Map. Available at: http://maps.cga.harvard.edu:8080/Hospital/. Accessed 07/12, 2012.

7. van Klei WA, Hoff RG, van Aarnhem EE, Simmermacher RKJ, Regli LPE, Kappen TH, et al. Effects of the introduction of the WHO "Surgical Safety Checklist" on in-hospital mortality: A cohort study. *Annals of Surgery* 2012;255(1):44-9.

For peer review only - http://bmjopen.bmj.com/site/about/guidelines.xhtml

05/07/2013

8. Fourcade A, Blache J-, Grenier C, Bourgain J-, Minvielle E. Barriers to staff adoption of a surgical safety checklist. *BMJ Quality and Safety* 2012;21(3):191-7.

9. Levy SM, Senter CE, Hawkins RB, Zhao JY, Doody K, Kao LS, et al. Implementing a surgical checklist: More than checking a box. *Surgery (United States)* 2012;152(3):331-6.

10. Bosk CL, Dixon-Woods M, Goeschel CA, Pronovost PJ. The art of medicine. Reality check for checklists. *The Lancet* 2009;374(9688):444-5.

11. Dixon-Woods M, Suokas A, Pitchforth E, Tarrant C. An ethnographic study of classifying and accounting for risk at the sharp end of medical wards. *Soc Sci Med* 2009; Aug;69(3):362-9.

12. McCarthy JC, Wright PC, Monk AF, Watts LA. Concerns at work: Designing useful procedures. *Human-Computer Interaction* 1998;13:433-57.

13. Vats A, Vincent CA, Nagpal K, Davies RW, Darzi A, Moorthy K. Practical challenges of introducing WHO surgical checklist: UK pilot experience. *BMJ* 2010;340.

14. Conley DM, Singer SJ, Edmondson L, Berry WR, Gawande AA. Effective surgical safety checklist implementation. *J Am Coll Surg* 2011;212(5):873-9.

15. Whyte S, Cartmill C, Gardezi F, Reznick R, Orser BA, Doran D, et al. Uptake of a team briefing in the operating theatre: A Burkean dramatistic analysis. *Social Science and Medicine* 2009;69(12):1757-66.

16. Safe Surgery in Africa. Available at: http://www.who.int/patientsafety/news/sssl_articles/en/index.html. Accessed 7/7, 2012.

17. Dixon-Woods M. What can ethnography do for quality and safety in health care?. *Qual Saf Health Care* 2003; Oct;12(5):326-7.

18. Attride-Stirling J. Thematic networks: An analytic tool for qualitative research. *Qualitative Research* 2001;1(3):385,405; 385.

19. Charmaz K. *Constructing grounded theory: A practical guide through qualitative analysis.* London: Sage; 2006.

20. WHO World Alliance for Patient Safety. Implementation manual WHO Surgical Safety Checklist (first edition). ; 2008. Report No.: WHO/IER/PSP/2008.05.

21. Hsia RY, Mbembati NA, MacFarlane S, Kruk ME. Access to emergency and surgical care in sub-Saharan Africa: The infrastructure gap. *Health Policy Plan* 2012;27(3):234-44.

22. Deckelbaum DL, Ntakiyiruta G, Liberman AS, Razek T, Kyamanywa P. Augmenting surgical capacity in resource-limited settings. *The Lancet* 2011;.

23. Funk LM, Weiser TG, Berry WR, Lipsitz SR, Merry AF, Enright AC, et al. Global operating theatre distribution and pulse oximetry supply: An estimation from reported data. *The Lancet* 2010;376(9746):1055-61.

For peer review only

05/07/2013

24. De Vries EN, Prins HA, Crolla RMPH, Den Outer AJ, Van Andel G, Van Helden SH, et al. Effect of a comprehensive surgical safety system on patient outcomes. *N Engl J Med* 2010;363(20):1928-37.

25. Whyte S, Lingard L, Espin S, Baker GR, Bohnen BA, Orser B, et al. Paradoxical effects of interprofessional briefings on OR team performance. *Cognition, Technology and Work* 2008;10(4):287-94.

26. Wilson D. Who Guards the Guardians? Ian Kennedy, Bioethics and the 'Ideology of Accountability' in British Medicine. *Social History of Medicine* 2011; July 01;.

27. Aiken AM, Karuri DM, Wanyoro AK, Macleod J. Interventional studies for preventing surgical site infections in sub-Saharan Africa - A systematic review. *International Journal of Surgery* 2012;10(5):242-9.

28. Siddiqi K, Newell J, Robinson M. Getting evidence into practice: What works in developing countries?. *International Journal of Quality in Health Care* 2005;17:447-54.

29. Edmondson AC. Speaking up in the operating room: How team leaders promote learning in interdisciplinary action teams. *Journal of Management Studies* 2003;40(6):1419-52.

30. McCulloch P, Rathbone J, Catchpole K. Interventions to improve teamwork and communications among healthcare staff. *British Journal of Surgery* 2011;98(4):469-79.

31. Ider B-, Adams J, Morton A, Whitby M, Muugolog T, Lundeg G, et al. Using a checklist to identify barriers to compliance with evidence-based guidelines for central line management: A mixed methods study in Mongolia. *International Journal of Infectious Diseases* 2012;16(7):e551-7.

32. Kotagal M, Lee P, Habiyakare C, Dusabe R, Kanama P, Epino HM, et al. Improving quality in resource poor settings: Observational study from rural Rwanda. *BMJ* 2009;339:1311-3.

33. Walker IA, Merry AF, Wilson IH, McHugh GA, O'Sullivan E, Thoms GM, et al. Global oximetry: An international anaesthesia quality improvement project. *Anaesthesia* 2009;64(10):1051-60.

For peer review only

For peer review only - http://bmjopen.bmj.com/site/about/guidelines.xhtml
